# Molecular mechanisms involved in the IL-6-mediated upregulation of indoleamine 2,3-dioxygenase 1 (IDO1) expression in the chorionic villi and decidua of women in early pregnancy

**DOI:** 10.1186/s12884-022-05307-5

**Published:** 2022-12-31

**Authors:** Rui Wang, Shuyun Zhao, Xiaojuan Chen, Ziwen Xiao, Xinghui Wen, Xingming Zhong, Shixiang Li, Hui Cheng, Guanyou Huang

**Affiliations:** 1 Reproductive Medicine Center, Department of Obstetrics and Gynecology, The affiliated Hospital of Guizhou Medical University, Guiyang, 550004 Guizhou Province China; 2 Department of Hyperbaric Oxygen Chamber, The Affiliated Hospital of Guizhou Medical University, Guiyang, 550004 Guizhou Province China; 3 Department of Obstetrics and Gynecology, The Affiliated Hospital of Guizhou Medical University, Guiyang, 550004 Guizhou Province China; 4 Family Planning Research Institute of Guangdong, Guangzhou, 510600, Guangdong Province China

**Keywords:** IL-6, IDO1, SOCS3, maternal-foetal tolerance

## Abstract

**Background:**

IL-6 induces the upregulation of indoleamine 2,3-dioxygenase (IDO1) at the maternal-foetal interface, but the regulation mechanisms of IDO1 by IL-6 at this interface have not been fully understood.

**Methods:**

Western blotting, qRT–PCR and/or immunohistochemistry were employed to measure the expression of IDO1, IL-6, SHP-1/2, SOCS3 and STAT3/p (STAT3 and pSTAT3) in tissues of chorionic villi and decidua (TCVD) in vivo and in cultured TCVD that were treated with IL-6 in the presence or absence of an IL-6 inhibitor.

**Results:**

Mutually positive relationships among the protein levels of IL-6, IDO1, SHP-1/2 and STAT3/p was observed, and the expression of IDO1, SHP-1/2 and STAT3/p was increased in a dose-dependent manner in TCVD in vivo and in cultured TCVD treated with IL-6 at increasing concentrations (0–100 ng/ml). The level of IL-6 was negatively related to SOCS3 level in TCVD. The expression of SOCS3 was increased in a dose-dependent manner, and SOCS3 level was positively correlated with SHP-1, SHP-2 and STAT3/p level in cultured TCVD treated with 0–2 ng/ml IL-6; however, opposite results were observed after treatment with 2–100 ng/ml IL-6. The IL-6-induced upregulation of IDO1, SHP-1, SHP-2 and STAT3/p expression could be reversed, while the IL-6-induced upregulation of SOCS3 expression was exacerbated by Corylifol A.

**Conclusions:**

In normal pregnancy, IL-6 upregulates the expression of IDO1 by promoting SHP-1/2 expression via STAT3/p and simultaneously negatively regulates the expression of SOCS3. High expression of IL-6 causes the upregulation of IDO1 expression and the downregulation of SOCS-3 expression, which may be beneficial for maintaining immunological tolerance.

**Supplementary Information:**

The online version contains supplementary material available at 10.1186/s12884-022-05307-5.

## Background

Indoleamine 2,3-dioxygenase (IDO1) is an enzyme that is involved in tryptophan catabolism; IDO1 catalyses the first and rate-limiting step of tryptophan oxidative degradation and may lead to the production of a series of downstream metabolites called kynurenines [1]. During pregnancy, IDO1 is chiefly expressed in the trophoblast [2, 3], and has been shown to be a major protein that helps protect the foetus from the attack of maternal immune system by inhibiting T-lymphocyte responses through tryptophan consumption and tryptophan catabolism defects [4–7].

After stimulation, CD4+ T helper cells may release various cytokines that are classified as T helper (Th)1-, Th2- and Th17-type cytokines. Th1-type cytokines may be related to immune rejection, and Th2-type cytokines may be linked to maternal-foetal immunotolerance and successful pregnancy. Changes in the Th1/Th2/Th17 ratio appear to affect pregnancy outcomes [8]. Interleukin 6 (IL-6), which is a Th2-type cytokine, exerts extensive effects on inflammation, immune responses, and haematopoiesis [9]. Our previous report showed that IL-6 can induce the upregulation of IDO1 in the chorionic villi and decidua of women in early pregnancy, which suggests that Th2-type cytokines (such as IL-6) induce maternal-foetal tolerance via the upregulation of IDO1 [10]. In this study, we investigated the molecular mechanism by which IL-6 upregulates IDO1 expression at the maternal-foetal interface.

## Methods

### Patients and samples

Thirty-eight healthy pregnant women (age, 27.20 ± 5.32 years; gestational age, 57.62 ± 8.43 days) who underwent legal pregnancy termination at the Affiliated Hospital of Guizhou Medical University, Guiyang, from Jan 2019 to October 2020 were enrolled in this study. All the enrolled women exhibited normal embryonic development according to ultrasound examination. Subjects with a history of abnormal reproduction and chronic diseases associated with chronic hypertension, kidney disease, or diabetes were excluded from the study. The samples were collected and prepared as described previously [11], namely, the gestational tissues (decidua and chorionic villi) were collected aseptically during dilatation and curettage. The collected tissue mixtures were grossly separated into decidua and chorionic villi, which were then immediately placed into 0.1 M sterile phosphate-buffered saline (PBS, pH = 7.2) and transported to the laboratory on ice within 10 min of collection. Then, the samples were washed with PBS to remove red blood cells. Tissue samples of chorionic villi and decidua were either preserved at − 80 °C prior to Western blotting analysis or cut into small blocks (no more than 1 mg wet weight) for culture. Tissue samples used for culture were processed within 40 min of collection.

### Tissue culture

The tissue culture samples were collected and prepared as described in a previous study [11]; the tissues were cut into small blocks, washed twice with F-12/DMEM (Invitrogen, USA), centrifuged (1500 rpm, 5 min), and then cultured in F-12/DMED (Invitrogen) supplemented with 10% FBS (Gibco; Australia) and 1% penicillin–streptomycin (Invitrogen). Tissue explants were then placed into a CO_2_ incubator (37 °C, 5% CO_2_, approximately 0.1 g explants) (Thermo Fisher Scientific, USA) and cultured with 2 mL culture medium. IL-6 (PEPROTECH, USA) was diluted in 0.1% BSA (Sigma–Aldrich, USA) and PBS (Sigma–Aldrich); Corylifol A (MedChemExpress, USA) was diluted with 10% DMSO (Sigma–Aldrich), 40% PEG300 (MedChemExpress), 5% Tween-80 (Sigma–Aldrich) and 45% saline (Sigma–Aldrich).

### Western blotting analysis

The procedure for Western blotting analysis was reported previously [11]. Briefly, tissues from chorionic villi and decidua or cultured tissue explants from chorionic villi and decidua were placed into a liquid nitrogen-containing mortar, and then, the samples were homogenised into powder. Then, radioimmune precipitation assay buffer (Sigma–Aldrich) supplemented with protease inhibitors (P8340; Sigma–Aldrich) was added and incubated for 30 min at 4 °C, followed by two rounds of centrifugation (13,000 rpm, 20 min, 4 °C). Next, the supernatants were loaded onto gels and subjected to SDS polyacrylamide gel electrophoresis (SDS–PAGE) to separate the proteins. Then, the proteins were transferred onto polyvinylidene fluoride (PVDF) membranes (PerkinElmer, USA). The blots were incubated (3 h, room temperature) with the following primary antibodies: rabbit anti-human IDO1 monoclonal antibody; anti-human SOCS3 (L210) antibody; anti-human SHP-1 antibody; anti-human SHP-2 antibody; and anti-STAT3 and anti-pSTAT3 antibodies (all used at a dilution of 1:3000 and obtained from Cell Signaling Technology, USA) or rabbit anti-human glyceraldehyde-3-phosphate dehydrogenase (GAPDH) polyclonal antibody (1:6000; GeneTex, CA, USA). Then, the blots were rinsed with tris-buffered saline containing Tween-20. After that, the secondary horseradish peroxidase (HRP)-conjugated goat anti-rabbit immunoglobulin G antibody (Cell Signaling Technology, USA) was added and incubated. The proteins were visualized using the ECL kit (PerkinElmer, USA), followed by autoradiography.

### Quantitative reverse transcription-polymerase chain reaction (qRT–PCR)

The procedure used for qRT–PCR was described previously [11]. Briefly, total mRNA was extracted from the cultured tissue explants of chorionic villi and decidua using an RNA extraction kit (Axygen, USA). Next, cDNA was synthesized from 1 μg of total RNA using oligo dT and Quant Reverse Transcriptase (TaKaRa, Japan). SYBR Green (TaKaRa, Japan) was used for real-time quantitative PCR following the manufacturer’s instructions. The primers specific for the human SOCS3 and IDO1 genes were designed according to the gene sequences and then synthesized by Thermo Fisher. The 5′ oligonucleotide primers used for amplification were as follows: for human SOCS3, sense 5′-GCTCCAAGAGCGAGTACCAG-3′, anti-sense 5′-CTGTCGCGGATCAGAAAGGT-3′; for human IDO1, sense 5′-ATATGCCACCAGCTCACAGG-3′, anti-sense 5′-AGCTTTCACACAGGCGTCAT-3′. The PCR conditions were 95 °C for 30 s and then 40 cycles of 95 °C for 5 s, 60 °C for 34 s, 95 °C for 15 s and 61 °C for 30 s. Gene relative expression levels were calculated using the 2^-ΔΔCT^ method.

### Immunohistochemistry

The procedure used for immunohistochemistry were described previously [12]. Briefly, tissue specimens were paraffin-embedded, and each sample was continuously sectioned (5 μm). After dewaxing and rehydration, the sections were immersed in ethylenediaminetetraacetic acid solution and boiled in an electric pressure cooker (3 min) for antigen retrieval. After cooling at room temperature, endogenous peroxidase activity was quenched using 3% hydrogen peroxide (H_2_O_2_). The sections were washed with 0.1 MPBS (pH = 7.4) after each step of the immunostaining process. The primary antibodies (mouse anti-human IDO1, mouse anti-human SOCS3, mouse anti-human IL-6 monoclonal antibodies; cat. Nos. ab55305, ab14939 and ab216492, respectively; Abcam, Cambridge, UK) were diluted with antibody dilution buffer (Beijing Zhongshan Golden Bridge Biotechnology Co., Ltd., Beijing, China) at dilutions of 1:200, 1:120 and 1:200 for the IDO1, SOCS3 and IL-6 antibodies, respectively. Then, the sections were incubated (overnight, 4 °C) with the primary antibodies. Next, the sections were incubated (30 min, 37 °C) with a rabbit anti-mouse HRP antibody (1:1000; cat. no. K5007; Dako; Agilent Technologies, Inc., Santa Clara, CA, USA). After washing with PBS (pH = 7.4) 3 times, the sections were developed using 3,3′-diaminobenzidine (DAB) solution from a DAB kit (Dako; Agilent Technologies, Inc.), counterstained with haematoxylin and dehydrated. Then, sections were mounted with coverslips. The immunohistochemical images were captured using an Olympus microscope (Olympus, Tokyo, Japan).

### Statistical analyses

All the statistical analyses were performed using the GraphPad statistical software package, version 6.0 (GraphPad Software, San Diego, CA) and SPSS (IBM, USA). The protein expression data from the Western blotting analyses were analysed using Pearson’s correlation analysis and the paired-samples *t* test. Significance was set to *P* < 0.05.

### Ethics approval

This study was conducted with strict compliance to the principles outlined in the Declaration of Helsinki. The study was approved by the Ethics Committee of the Affiliated Hospital of Guizhou Medical University (Guizhou, China).

## Results

### The relationship of IL-6 and IDO1 expression with SHP-1, SHP-2, STAT3, pSTAT3 and SOCS3 expression in chorionic villus and decidual tissues from women in early pregnancy

**Fig. 1:**
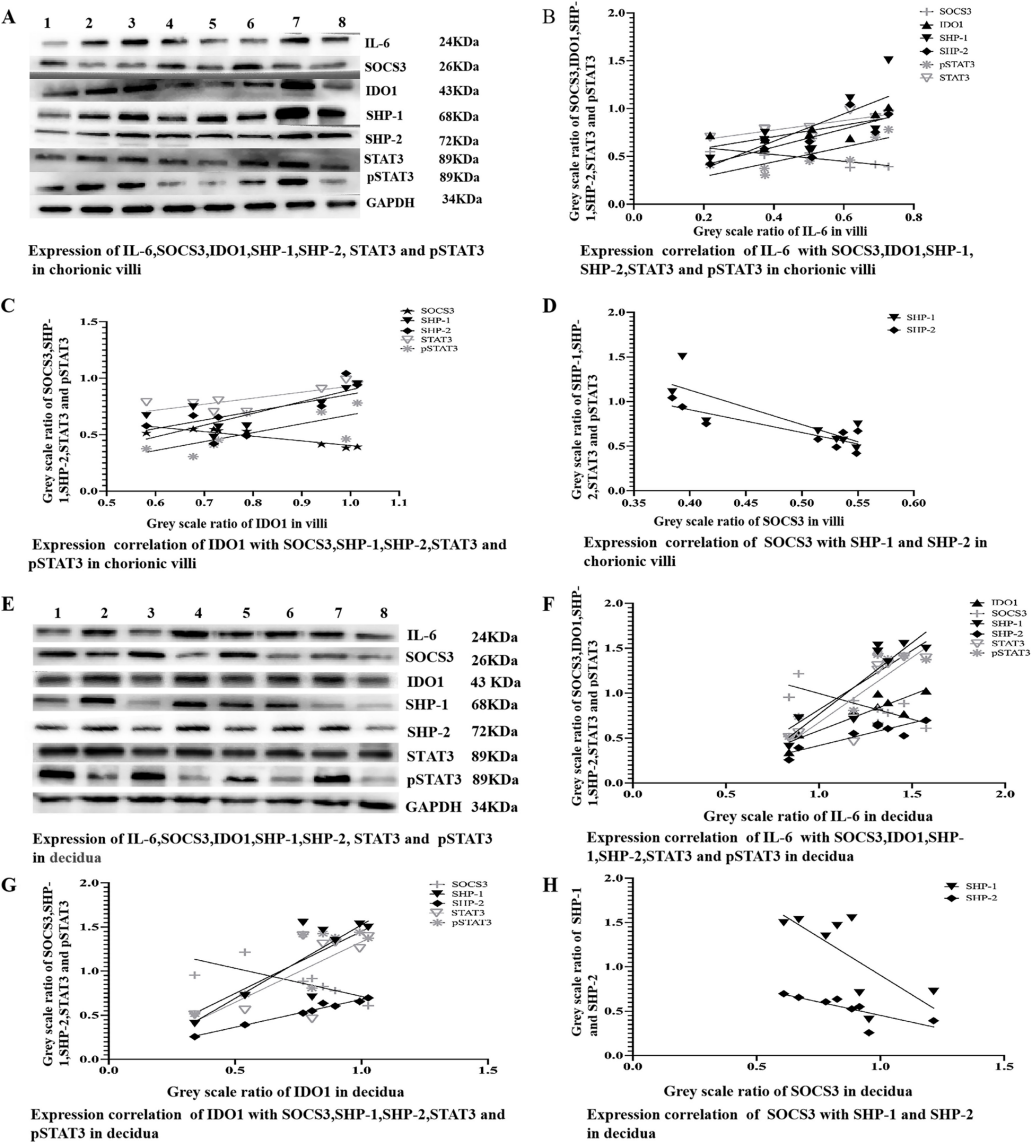
The expression of IL-6, IDO1, SHP-1, SHP-2, STAT3, pSTAT3 and SOCS3 and the correlation of these expression levels. **A**-**D** The expression of IL-6, IDO1, SHP-1, SHP-2, STAT3, pSTAT3, and SOCS3 and the correlation of these expression levels in chorionic villi. A. The expression of IL-6, IDO1, SHP-1, SHP-2, STAT3, pSTAT3 and SOCS3 in chorionic villi, as measured by Western blotting analysis. **B**, **C** and **D** The correlation of protein expression levels of IL-6, IDO1, SHP-1, SHP-2, STAT3, pSTAT3, and SOCS3 in chorionic villi. Data represent a portion of each sample from thirty-eight healthy pregnant women. Labels 1–8 represent different samples from healthy pregnant women. **B** The correlation coefficients (r) of the protein expression levels of IL-6 and IDO1, SHP-1, SHP-2, STAT3, pSTAT3 and SOCS3 were 0.735, 0.72, 0.77, 0.8047, 0.767, and − 0.844, respectively; the *P* values for the correlation of the protein expression levels between IL-6 and IDO1, SHP-1, SHP-2, STAT3, pSTAT3, and SOCS3 were 0.0377, 0.0400, 0.0241, 0.0160, 0.0262, and 0.0083, respectively. **C** The correlation coefficients (r) of the protein expression levels of IDO1 and SHP-1, SHP-2, STAT3, pSTAT3 and SOCS3 were 0.719, 0.7746, 0.78, 0.7147 and − 0.8923, respectively; the *P* values for correlation of the protein expression levels of IDO1 and SHP-1, SHP-2, STAT3, pSTAT3, and SOCS3 were 0.0443, 0.0240, 0.0198, 0.0463, and 0.0029, respectively. **D** The correlation of the protein expression levels of SOCS3 and SHP-1/2 was − 0.824/− 0.8773, respectively; the *P* value for the correlation of the protein expression levels of SOCS3 and SHP-1/2 was 0.0117/0.0042, respectively. **E-H** The expression of IL-6, IDO1, SHP-1, SHP-2, STAT3, pSTAT3, and SOCS3 and the correlation of these expression levels in decidua. **E** The expression of IL-6, IDO1, SHP-1, SHP-2, STAT3, pSTAT3 and SOCS3 in the decidua, as measured by Western blotting analysis. **F**, **G** and **H** The correlation of the protein expression levels of IL-6, IDO1, SHP-1, SHP-2, STAT3, pSTAT3 and SOCS3 in decidua. The data represent a portion of each sample from thirty-eight healthy pregnant women. Labels 1–8 represent different samples from healthy pregnant women. **F** The correlation coefficients (r) of the protein expression levels of IL-6 and IDO1, SHP-1, SHP-2, STAT3, pSTAT3, and SOCS3 were 0.8914, 0.8947, 0.8846, 0.8694, 0.8949, and − 0.7950, respectively; the *P* values for the correlation of the protein expression levels of IL-6 and IDO1, SHP-1, SHP-2, STAT3, pSTAT3 and SOCS3 were 0.0029, 0.0027, 0.0035, 0.0050, 0.0027 and 0.0183, respectively. **G** The correlation coefficients (r) of the protein expression levels of IDO1 and SHP-1, SHP-2, STAT3, pSTAT3 and SOCS3 were 0.834, 0.9890, 0.7359, 0.8527, and − 0.7896, respectively; the *P* values for correlation of the protein expression levels of IDO1 and SHP-1, SHP-2, STAT3, pSTAT3, and SOCS3 were 0.010, < 0.0001, 0.0374, 0.0071, and 0.0198, respectively. **H** The correlation of the protein expression levels of SOCS3 and SHP-1/2 was − 0.712/− 0.7665, respectively; the *P* value for correlation of the protein expression levels of SOCS3 and SHP-1/2 was 0.0451/0.0265, respectively. The greyscale ratios of IL-6 and IDO1, SOCS3, SHP-1, SHP-2, STAT3 and pSTAT3 and the expression levels of IL-6 and IDO1, SOCS3, SHP-1/2, STAT3 and pSTAT3 were assessed based on the ratios of the greyscale value of the IL-6 and IDO1, SOCS3, SHP-1, SHP-2, STAT3 and pSTAT3 bands to that of the GAPDH band. IDO1, indoleamine 2,3-dioxygenase; SOCS3, suppressors of cytokine signalling 3; STAT3, signal transducer and activator of transcription 3; pSTAT3, phosphorylated STAT3; SHP-1/2, protein tyrosine phosphatase-1/2; IL-6, interleukin-6. GAPDH, glyceraldehyde 3-phosphate dehydrogenase

**Fig. 2:**
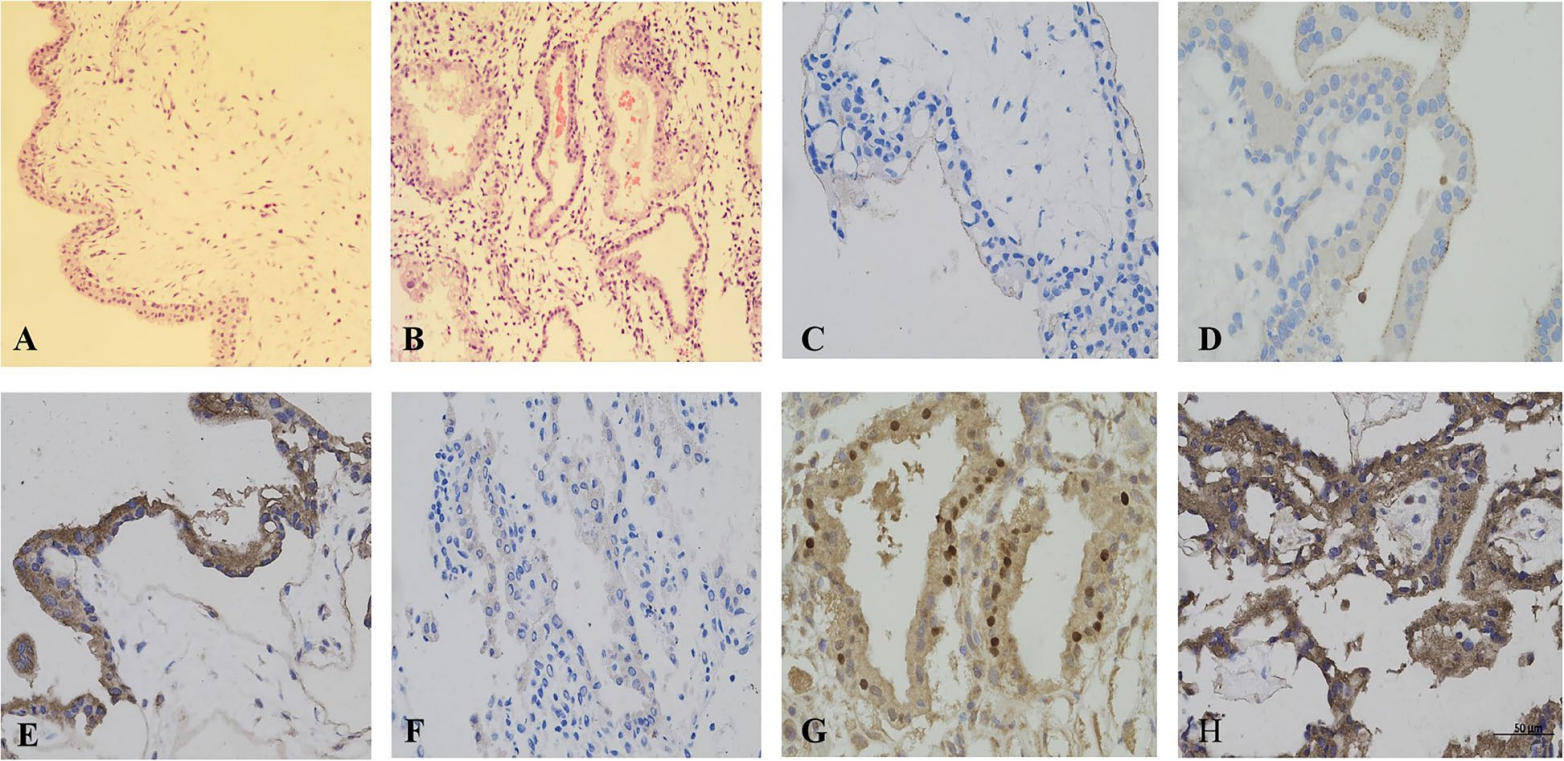
Photomicrograph of (**A**) chorionic villi and (**B**) decidua after haematoxylin and eosin (H&E) staining (200x magnification). The data represent a portion of each sample from thirteen healthy pregnant women. Staining for IL-6, IDO1 and SOCS3 showed that these proteins were scattered in syncytiotrophoblast of the chorionic villi (**C**-**E**) and extravillous cytotrophoblasts of decidua (**F**-**H**), respectively (400x magnification). Images were taken from the same chorionic villi (**A**, **C**-**E**) and decidua (**B**, **F**-**H**) sample. IL-6 (**C**, **F**) was present in the cytoplasm and cytomembrance and was stained yellow or light yellow, IDO1 (**D**, **G**) was localized in the cytoplasm and was stained yellow or brown yellow, SOCS3 was present in both the cytoplasm and nucleus was stained yellow or brown yellow (**E**, **H**)

To investigate the relationship of IL-6 and IDO1 expression with the expression of SHP-1, SHP-2, STAT3, pSTAT3, and SOCS3 at the maternal-foetal interface, chorionic villus and decidual tissues were homogenized into powders and then lysed for Western blotting analysis. The results revealed that IL-6, IDO1, SHP-1, SHP-2, STAT3, and pSTAT3 were expressed in both the chorionic villi and decidua (Fig. 1A, E); IL-6 expression was positively correlated with IDO1, SHP-1, SHP-2, STAT3, and pSTAT3 expression (Fig. 1A, B, E, F); IDO1 expression was positively correlated with SHP-1, SHP-2, STAT3, and pSTAT3 expression (Fig. 1A, C, E, G); and SOCS3 expression was negatively correlated with IL-6, SHP-1, SHP-2, STAT3, and pSTAT3 expression (Fig. 1A-D; E-H). The Western blotting analysis results from chorionic villus and decidual tissues demonstrated that there was a positive relationship between IL-6 expression and IDO1, SHP-1, SHP-2, STAT3, and pSTAT3 expression and a negative relationship between SOCS3 expression and IL-6, IDO1, SHP-1, SHP-2, STAT3, and pSTAT3 expression; these results suggested that IL-6 may simultaneously upregulate the expression of IDO1, SHP-1, SHP-2, STAT3, and pSTAT3 and that high expression of IL-6 might be closely associated with low expression of SOCS3. As shown in the immunocytochemical staining results, IL-6, IDO1, and SOCS3 were expressed in the syncytiotrophoblasts and extravillous cytotrophoblasts of the chorionic villi and decidua, and IDO1 was present in the cytoplasm, IL-6 was localized in the cytoplasm and cytomembrane, and SOCS3was localized in both the cytoplasm and nucleus. The immunohistochemistry results were examined at × 400, and the H&E staining results were examined at × 200 magnification. Several representative photomicrographs are shown in Fig. 2.

### IL-6 increased IDO1, SOCS3, SHP-1, SHP-2, STAT3, and pSTAT3 expression in chorionic villi and decidua tissues

**Fig. 3:**
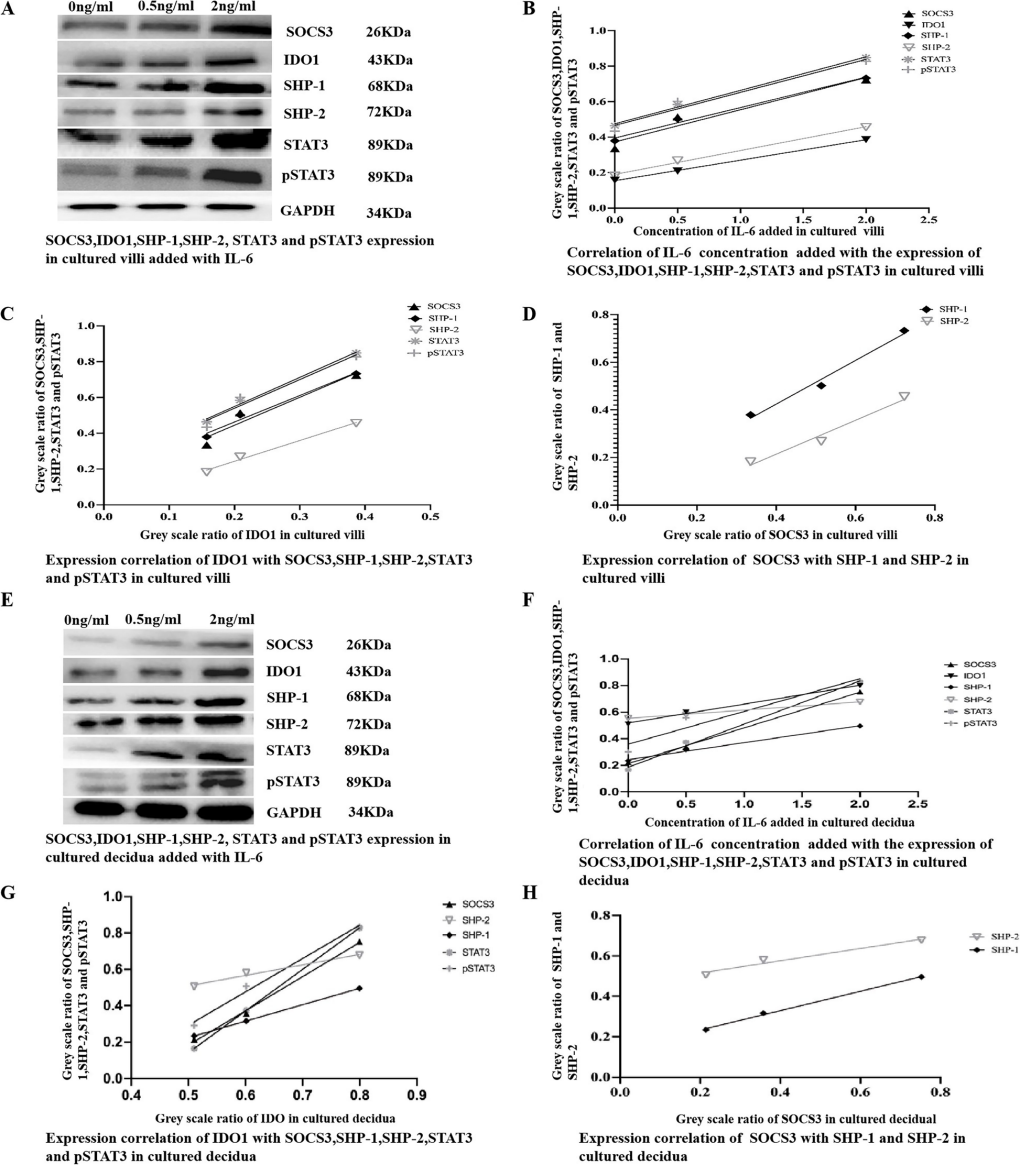
**A-D** The expression of IDO1, SHP-1, SHP-2, STAT3, pSTAT3 and SOCS3 in chorionic villi cultured with a low concentration of IL-6 (0–2 ng/ml) for 24 h and the correlation of these expression levels, as measured by Western blotting analysis. The expression of IDO1, SHP-1, SHP-2, STAT3, pSTAT3, and SOCS3 (**A**), the correlation of the IL-6 concentration added to chorionic villi cultures with the expression of IDO1, SHP-1, SHP-2, STAT3, pSTAT3, and SOCS3 (**B**), the correlation of IDO1 expression with the expression of SHP-1, SHP-2, STAT3, pSTAT3, and SOCS3 (**C**), and the correlation of SOCS3 expression with the expression of SHP-1 and SHP-2 (**D**) in chorionic villi cultured with low concentrations of IL-6 (0–2 ng/ml) for 24 h were evaluated and analysed. The data are presented as the mean ± SE of three similar experiments. B The correlation coefficients (r) between the IL-6 concentration added to chorionic villi cultures and the expression of IDO1, SHP-1, SHP-2, STAT3, pSTAT3 and SOCS3 were 0.992, 0.988, 0.951, 0.995, 0.962, and 0.9458, respectively; the *P* values for the correlations of the levels of IL-6 and IDO1, SHP-1, SHP-2, STAT3, pSTAT3 and SOCS3 were 0.0175, 0.0427, 0.0445, 0.0426, 0.0412 and 0.0447, respectively. **C** The correlation coefficients (r) of the protein expression levels of IDO1 and SHP-1, SHP-2, STAT3, pSTAT3 and SOCS3 were 0.9824, 0.9906, 0.9911, 0.9511 and 0.9327, respectively; the *P* value for correlation of the protein expression levels of IDO1 and SHP-1, SHP-2, STAT3, pSTAT3 and SOCS3 were 0.037, 0.0427, 0.0401, 0.0477, and 0.0413, respectively. **D** The correlation of the protein expression levels of SOCS3 and SHP-1/2 was 0.9883 and 0.9730, respectively; the *P* value for correlation of the protein expression levels of SOCS3 and SHP-1/2 was 0.0421 and 0.0352, respectively. **E-H** The expression of IDO1, SHP-1, SHP-2, STAT3, pSTAT3, and SOCS3 in decidua cultured with a low concentration of IL-6 (0–2 ng/ml) for 24 h and the correlations of these expression levels, as measured by Western blotting analysis. The expression of IDO1, SHP-1, SHP-2, STAT3, pSTAT3 and SOCS3 (**E**), the correlation of the IL-6 concentration added to the decidua cultures with the expression of IDO1, SHP-1, SHP-2, STAT3, pSTAT3 and SOCS3 (**F**), the correlation of IDO1 expression with the expression of SHP-1, SHP-2, STAT3, pSTAT3, and SOCS3 (**G**), and the correlation of SOCS3 expression with the expression of SHP-1 and SHP-2 (**H**) in decidua cultured with low concentrations of IL-6 (0–2 ng/ml) for 24 h were evaluated and analysed. The data are presented as the mean ± SE of three similar experiments. F The correlation coefficients (r) between the IL-6 concentration added to the decidua cultures and the expression of IDO1, SHP-1, SHP-2, STAT3, pSTAT3, and SOCS3 were 0.950, 0.951, 0.980, 0.950, 0.9345, and 0.991, respectively; the *P* values for the correlations of IL-6 expression and IDO1, SHP-1, SHP-2, STAT3, pSTAT3 and SOCS3 expression were 0.0452, 0.0446, 0.0285, 0.0450, 0.0412, and 0.0193, respectively. **G** The correlation coefficients (r) of the protein expression levels of IDO1 and SHP-1, SHP-2, STAT3, pSTAT3 and SOCS3 were 0.976, 0.9866, 0.9967, 0.987 and 0.989, respectively; the *P* value for correlation of the protein expression levels of IDO1 and SHP-1, SHP-2, STAT3, pSTAT3 and SOCS3 were 0.0007, 0.0437, 0.0124, 0.0002, and 0.0345, respectively. H The correlation of the protein expression levels of SOCS3 and SHP-1/2 was 0.973/9630, respectively; the *P* value for the correlation of the protein expression levels of SOCS3 and SHP-1/2 was 0.0331/0.0452. The greyscale ratios of IDO1, SOCS3, SHP-1, SHP-2, STAT3 and pSTAT3 and the expression levels of IDO1, SOCS3, SHP-1/2, STAT3 and pSTAT3 were assessed based on the ratios of the greyscale values of the IL-6 and IDO1, SOCS3, SHP-1, SHP-2, STAT3 and pSTAT3 bands to that of the GAPDH band. IDO1, indoleamine 2,3-dioxygenase; SOCS3, suppressors of cytokine signalling 3; STAT3, signal transducer and activator of transcription 3; pSTAT3, phosphorylated STAT3; SHP-1/2, protein tyrosine phosphatase-1/2; IL-6, interleukin-6; GAPDH, glyceraldehyde 3-phosphate dehydrogenase

**Fig. 4:**
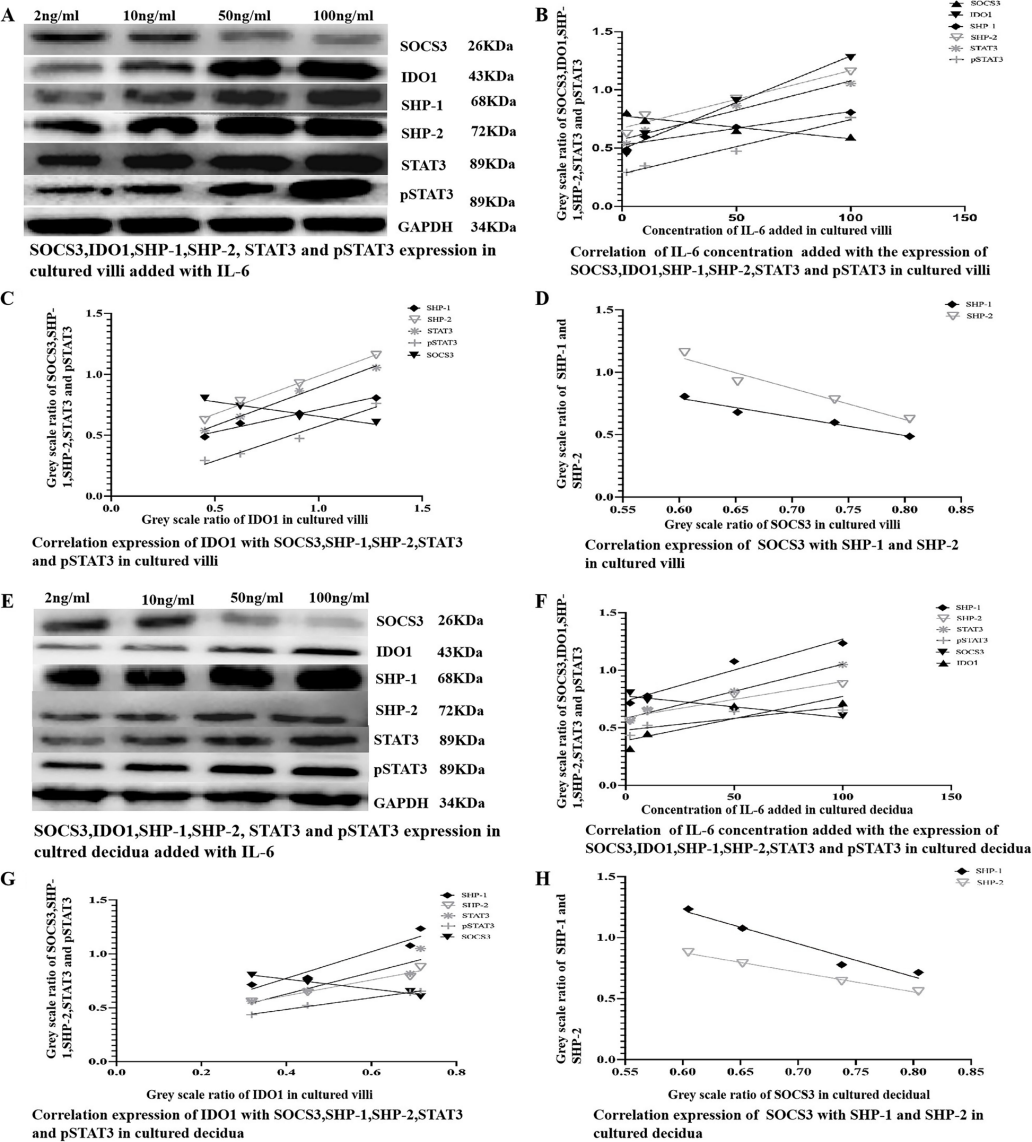
**A-D** The expression of IDO1, SHP-1, SHP-2, STAT3, pSTAT3 and SOCS3 in chorionic villi cultured with a high concentration of IL-6 (2–100 ng/ml) for 24 h and the correlations of these expression levels, as measured by Western blotting analysis. The expression of IDO1, SHP-1, SHP-2, STAT3, pSTAT3, and SOCS3 (**A**), the correlation of the IL-6 concentration added to chorionic villi cultures with the expression of IDO1, SHP-1, SHP-2, STAT3, pSTAT3 and SOCS3 (**B**), the correlation of IDO1 expression with the expression of SHP-1, SHP-2, STAT3, pSTAT3 and SOCS3 (**C**) and the correlation of SOCS3 expression with the expression of SHP-1 and SHP-2 (**D**) in chorionic villi cultured with high concentrations of IL-6 (2–100 ng/ml) for 24 h were evaluated and analysed. The data are presented as the mean ± SE of three similar experiments. **B** The correlation coefficients (r) between the IL-6 concentration added to chorionic villi cultures and the expression of IDO1, SHP-1, SHP-2, STAT3, pSTAT3 and SOCS3 were 0.9841, 0.9259, 0.9554, 0.9689, 0.9826, and − 0.8904, respectively; the *P* values for the correlations between IL-6 and IDO1, SHP-1, SHP-2, STAT3, pSTAT3 and SOCS3 were 0.0464, 0.0387, 0.0225, 0.0157, 0.0087 and 0.008, respectively. **C** The correlation coefficients (r) of the protein expression levels of IDO1 and SHP-1, SHP-2, STAT3, pSTAT3 and SOCS3 were 0.9776, 0.9981, 0.9928, 0.9672, and − 0.9458, respectively; the *P* values for correlation of the protein expression levels of IDO1 and SHP-1, SHP-2, STAT3, pSTAT3 and SOCS3 were 0.0112, 0.0041, 0.0036, 0.0165, and 0.0275, respectively. **D** The correlation of the protein expression levels of SOCS3 and SHP-1/2 was − 0.9670/− 0.9545, respectively; the *P* value for correlation of the protein expression levels of SOCS3 and SHP-1/2 was 0.0167/0.0230, respectively. **E-H** The expression and correlation of IDO1, SHP-1, SHP-2, STAT3, pSTAT3 and SOCS3 in decidua cultured with high concentrations of IL-6 (2–100 ng/ml) for 24 h, as measured by Western blotting analysis. The expression of IDO1, SHP-1, SHP-2, STAT3, pSTAT3 and SOCS3 (**E**), the correlation of IL-6 concentration added to the decidua cultures with the expression of IDO1, SHP-1, SHP-2, STAT3, pSTAT3 and SOCS3 (**F**), the correlation of IDO1 expression with the expression of SHP-1, SHP-2, STAT3, pSTAT3 and SOCS3 (**G**), and the correlation of SOCS3 expression with the expression of SHP-1 and SHP-2 (**H**) in decidua cultured with high-concentrations of IL-6 (2–100 ng/ml) for 24 h were evaluated and analysed. The data are presented as the mean ± SE of three similar experiments. **F** The correlation coefficients (r) between the IL-6 concentration added to the decidua cultures and the expression of IDO1, SHP-1, SHP-2, STAT3, pSTAT3 and SOCS3 were 0.8031, 0.9572, 0.9330, 0.9858, 0.7722 and − 0.8904, respectively; the *P* values for the correlations between IL-6 expression and IDO1, SHP-1, SHP-2, STAT3, pSTAT3 and SOCS3 expression were 0.0424, 0.0408, 0.0341, 0.0071, 0.0462 and 0.0462, respectively. **G** The correlation coefficients (r) of the protein expression levels of IDO1 and SHP-1, SHP-2, STAT3, pSTAT3 and SOCS3 were 0.9272, 0.9556, 0.8372, 0.9956 and − 0.9717, respectively; the *P* values for the correlation of the protein expression levels of IDO1 and SHP-1, SHP-2, STAT3, pSTAT3 and SOCS3 were 0.0371, 0.0224, 0.0420, 0.0022 and 0.0142, respectively. **H** The correlation of the protein expression levels of SOCS3 and SHP-1/2 was − 0.9575/− 0.993, respectively; the *P* value for correlation of the protein expression levels of SOCS3 and SHP-1/2 was 0.0215/0.0032, respectively. The greyscale ratios of IDO1, SOCS3, SHP-1, SHP-2, STAT3 and pSTAT3 and the expression levels of IDO1, SOCS3, SHP-1/2, STAT3 and pSTAT3 were assessed based on the ratios of the greyscale values of the IL-6 and IDO1, SOCS3, SHP-1, SHP-2, STAT3 and pSTAT3 bands to that of the GAPDH band. IDO1, indoleamine 2,3-dioxygenase; SOCS3, suppressors of cytokine signalling 3; STAT3, signal transducer and activator of transcription 3; pSTAT3, phosphorylated STAT3; SHP-1/2, protein tyrosine phosphatase-1/2; IL-6, interleukin-6; GAPDH, glyceraldehyde 3-phosphate dehydrogenase

**Fig. 5:**
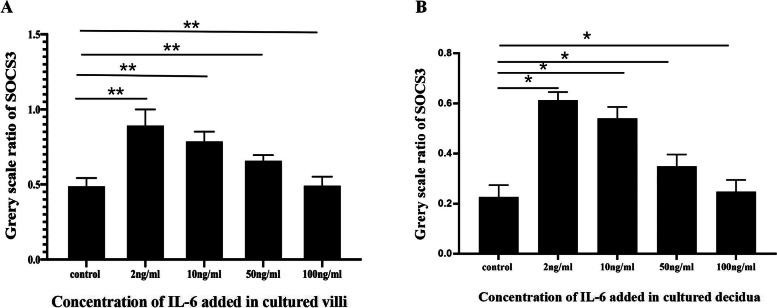
SOCS3 expression in chorionic villi (**A**) and decidua (**B**) cultured with high concentrations of IL-6 (2–100 ng/ml) for 24 h, as measured by Western blotting analysis. The data are presented as the mean ± SE of three similar experiments. *P < 0.001, 0.0072, 0.025, and > 0.05 compared with the groups treated with 0 ng/ml IL-6. ** *P* = 0.00562, 0.00942, 0.023, and > 0.05 compared with the groups treated with 0 ng/ml IL-6

**Fig. 6:**
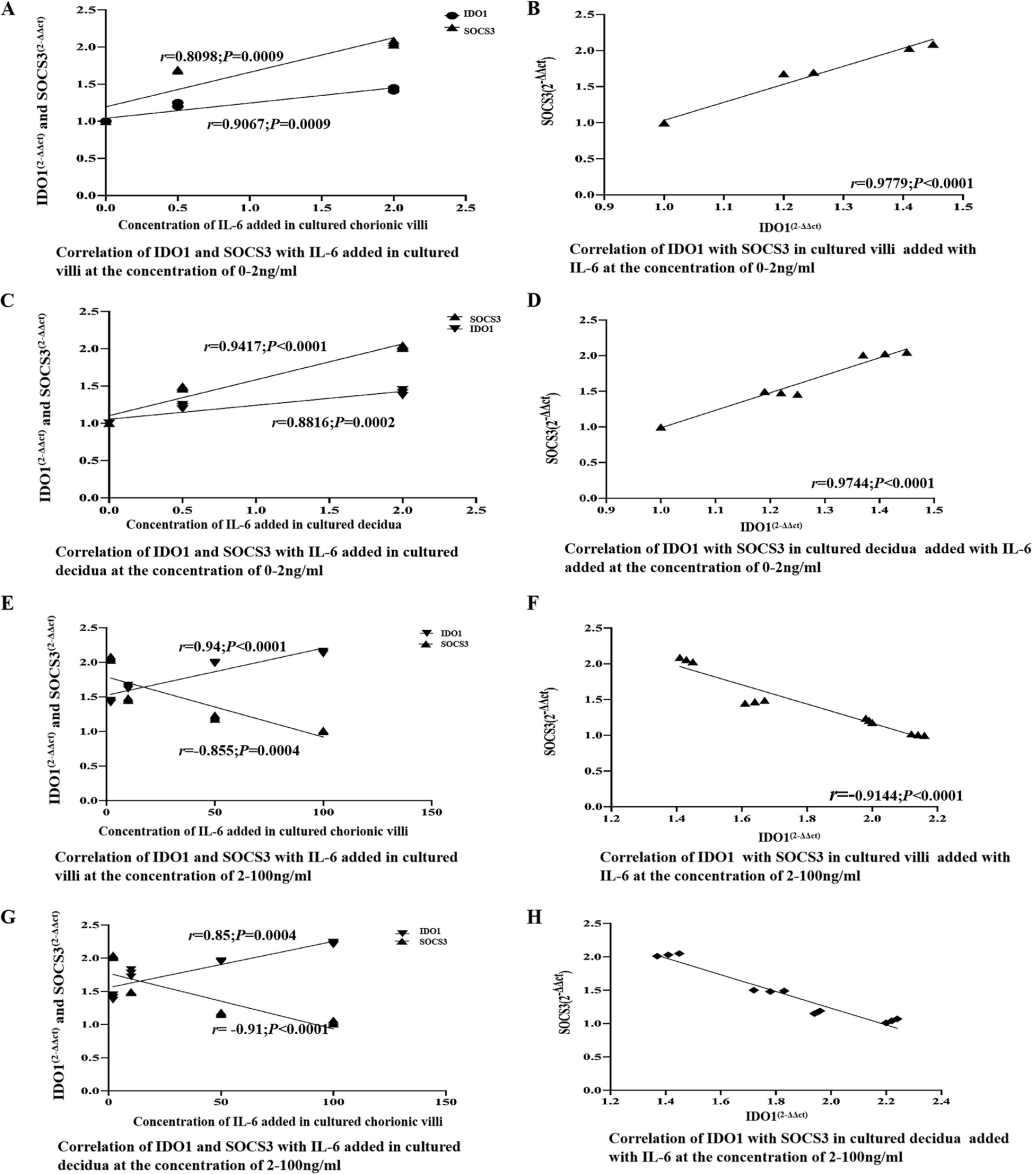
Correlation of IDO1 expression with SOCS3 expression in chorionic villi (**A** and **B**) and decidua (**C** and **D**) cultured with low concentrations of IL-6 (0–2 ng/ml) for 24 h, as measured by qRT-PCR. The correlation of the IL-6 concentration added to the cultures with the expression of SOCS3 and IDO1 mRNA in chorionic villi (**A**) and decidua (**C**) cultures and the correlation of IDO1 mRNA expression with SOCS3 mRNA expression in chorionic villi (**B**) and decidua (**D**) cultured with low concentrations of IL-6 (0–2 ng/ml) were evaluated and analysed. Correlation of IDO1 expression with SOCS3 expression in chorionic villi (**E** and **F**) and decidua (**G** and **H**) cultured with high concentration of IL-6 (2–100 ng/ml) for 24 h, as measured by qRT-PCR. The correlation of the IL-6 concentration added to the cultures with the expression of SOCS3 and IDO1 mRNA in cultured chorionic villi (**E**) and decidua (**G**) and the correlation of IDO1 mRNA expression with SOCS3 mRNA expression in chorionic villi (**F**) and decidua (**H**) cultured with high concentrations of IL-6 (2–100 ng/ml) were evaluated and analysed. The data are presented as the mean ± SE of three similar experiments. IDO1, indoleamine 2,3-dioxygenase; SOCS3, suppressors of cytokine signalling 3; IL-6, interleukin-6

To investigate the effect of IL-6 on the expression of IDO1, SHP-1, SHP-2, STAT3, pSTAT3, and SOCS3, tissue blocks from chorionic villi and decidua were incubated for 24 h in medium supplemented with IL-6 (0, 2, 10, 50, or 100 ng/ml). Subsequently, the tissue explants were lysed for Western blotting analysis and/or qRT–PCR to assess the expression of IDO1, SHP-1, SHP-2, STAT3, pSTAT3, and SOCS3. The Western blotting results demonstrated that the addition of increasing amounts of IL-6 to the culture medium at concentrations ranging from 0 ng/ml to 2 ng/ml increased the expression of IDO1, SHP-1, SHP-2, STAT3, pSTAT3, and SOCS3 in a dose-dependent manner (Fig. 3A, B, E, F)  in the cultured tissue explants of chorionic villi and decidua, IDO1 and SOCS3 expression was positively correlated with SHP-1, SHP-2, STAT3, and pSTAT3 expression (Fig. 3A, C, D, E, G, H) in the cultured tissues added with this low concentration of IL-6 (Fig. 3). The Western blotting further showed that the addition of increasing concentrations of IL-6 to the culture medium at concentrations ranging from 2 ng/ml to 100 ng/ml, increased the expression of IDO1, SHP-1, SHP-2, STAT3, pSTAT3, but decreased the expression of SOCS3 in a dose-dependent manner (Fig. 4A, B, E, F) in the cultured tissue explants of chorionic villi and decidua, IDO1 expression was positively, but SOCS3 expression was negatively, correlated with SHP-1, SHP-2, STAT3, and pSTAT3 expression (Fig. 4A, C, D, E, G, H) in the cultured tissues added with this high concentration of IL-6, and SOCS3 expression was decreased to the normal level when 100 ng/ml IL-6 was added to the culture medium (Fig. 5A-B). As shown by the qRT–PCR results, the addition of increasing amounts of IL-6 (0 ng/ml to 100 ng/ml) to the culture medium increased IDO1 mRNA expression in the tissue explants of chorionic villi (Additional file 1 Supplementary Table 1; Fig. 6A and E) and decidua (Additional file 1 Supplementary Table 2; Fig. 6C and G) in a dose-dependent manner. The addition of increasing concentrations of IL-6 to the culture medium increased SOCS3 mRNA expression in the tissue explants of chorionic villi (Additional file 1 Supplementary Table 3; Fig. 6A) and decidua (Additional file 1 Supplementary Table 4; Fig. 6C) in a dose-dependent manner, and SOCS3 mRNA expression was positively correlated with IDO1 mRNA expression in the tissue explants of chorionic villi (Additional file 1 Supplementary Table 3; Fig. 6B) and decidua (Additional file 1 Supplementary Table 4; Fig. 6D) after the addition of IL-6 at concentrations from 0 ng/ml to 2 ng/ml to the culture medium. The qRT–PCR results also showed that the addition of increasing amounts of IL-6 to the culture medium decreased SOCS3 mRNA expression in the tissue explants of chorionic villi (Additional file 1 Supplementary Table 3; Fig. 6 E) and decidua (Additional file 1 Supplementary Table 4; Fig. 6G) in a dose-dependent manner after the addition of IL-6 at concentrations from 2   ng/ml to 100 ng/ml to the culture medium  ; these levels were restored to normal levels when 100 ng/ml IL-6 was added to the culture medium (Additional file 1 Supplementary Table 3, Fig. 6E; Additional file 1 Supplementary Table 4, Fig. 6G). SOCS3 mRNA expression was negatively correlated with IDO1 mRNA expression in the tissue explants of chorionic villi (Additional file 1 Supplementary Table 3; Fig. 6F) and decidua (Additional file 1 Supplementary Table 4; Fig. 6H) that were cultured in medium supplemented with IL-6 at concentrations ranging from 2 ng/ml to 100 ng/ml. As shown by the Western blotting analysis of chorionic villus and decidual tissues cultured in medium supplemented with different concentrations of IL-6 (0–100 ng/ml), low concentrations (0–2 ng/ml) of IL-6 may simultaneously upregulate the expression of IDO1, SHP-1, SHP-2, STAT3, pSTAT3, and SOCS3 in a dose-dependent manner, while high concentrations of IL-6 (2–100 ng/ml) may upregulate the expression of IDO1, SHP-1, SHP-2, STAT3, and pSTAT3 and simultaneously reduce SOCS3 expression in a dose-dependent manner. The qRT–PCR results were consistent with the Western blotting results. Namely, a low concentration of IL-6 (0–2 ng/ml) may upregulate IDO1 and SOCS3 expression, while a high concentration of IL-6 (2–100 ng/ml) may reduce SOCS3 expression.

### Corylifol a decreased the IL-6-dependent regulation of IDO1 and SOCS3 expression

**Fig. 7:**
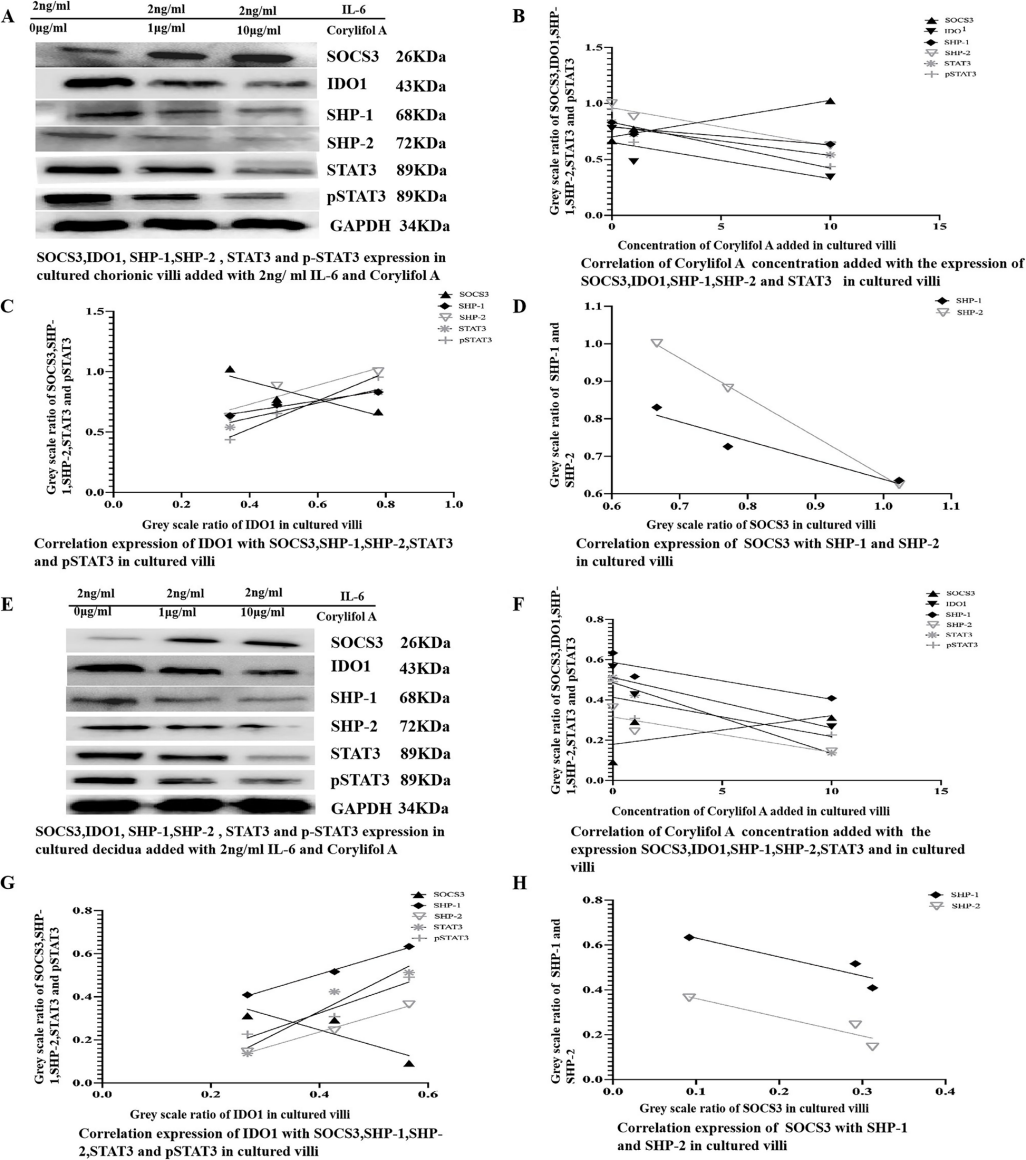
**A-D** The expression of IDO1, SHP-1, SHP-2, STAT3, pSTAT3, and SOCS3 in chorionic villi cultured with IL-6 and different concentrations of Corylifol A  for 24 h and the correlation of these expression levels, as measured by Western blotting analysis. The expression of IDO1, SHP-1, SHP-2, STAT3, pSTAT3 and SOCS3 (**A**) the correlation of the Corylifol A concentration added to chorionic villi cultures with the expression of IDO1, SHP-1, SHP-2, STAT3, pSTAT3 and SOCS3 (**B**) the correlation of IDO1 expression with of SHP-1, SHP-2, STAT3, pSTAT3 and SOCS3 expression (**C**) and the correlation of SOCS3 expression with the expression of SHP-1 and SHP-2 (**D**) in chorionic villi cultured with 2 ng/ml IL-6 and different concentrations of Corylifol (0, 1, or 10 μg/ml) for 24 h were evaluated and analysed. The data are presented as the mean ± SE of three similar experiments. **B** The correlation coefficients (r) between the Corylifol A concentration added to chorionic villi cultures and the expression of IDO1, SHP-1, SHP-2, STAT3, pSTAT3 and SOCS3 were − 0.96, − 0.88, − 0.95, − 0.9246, and 0.888, respectively; the *P* values for the correlation of IL-6 expression and IDO1, SHP-1, SHP-2, STAT3, pSTAT3 and SOCS3 expression were 0.01256, 0.0302, 0.014, 0.017 and 0.0408, respectively. **C** The correlation coefficients (r) of the protein expression levels of IDO1 and SHP-1, SHP-2, STAT3, STAT3 and SOCS3 were 0.972, 0.8361, 0.8735, 0.9865 and − 0.8165, respectively; the *P* value for correlation of the protein expression levels of IDO1 and SHP-1, SHP-2, STAT3, pSTAT3 and SOCS3 was 0.0116, 0.026, 0.023, 0.007 and 0.028, respectively. **D** The correlation of the protein expression levels of SOCS3 and SHP-1/2 was − 0.9259/− 0.9983, respectively; the *P* value for correlation of the protein expression levels of SOCS3 and SHP-1/2 was 0.01755/0.0016, respectively. The greyscale ratios of IDO1, SOCS3, SHP-1, SHP-2, STAT3 and pSTAT3 and the expression levels of IDO1, SOCS3, SHP-1/2, STAT3 and pSTAT3 were assessed based on the ratios of the greyscale values of the IDO1, SOCS3, SHP-1, SHP-2, STAT3 and pSTAT3 bands to that of the GAPDH band. IDO1, indoleamine 2,3-dioxygenase; SOCS3, suppressors of cytokine signalling 3; STAT3, signal transducer and activator of transcription 3; pSTAT3, phosphorylated STAT3; SHP-1/2, protein tyrosine phosphatase-1/2; IL-6, interleukin-6; GAPDH, glyceraldehyde 3-phosphate dehydrogenase. **E**-**H** The expression of IDO1, SHP-1, SHP-2, STAT3, pSTAT3 and SOCS3 in decidua cultured with IL-6 and different concentrations of Corylifol A (2–100 ng/ml) for 24 h and the correlation of these expression levels, as measured by Western blotting analysis. The expression of IDO1, SHP-1, SHP-2, STAT3, pSTAT3 and SOCS3 (**E**), the correlation of Corylifol A concentration added to decidua cultures with the expression of IDO1, SHP-1, SHP-2, STAT3, pSTAT3 and SOCS3 (**F**), the correlation of IDO1 expression with the expression of SHP-1, SHP-2, STAT3, pSTAT3 and SOCS3 (**G**), and the correlation of SOCS3 expression with the expression of SHP-1 and SHP-2 (**H**) in decidua cultured with 2 ng/ml IL-6 and different concentrations of Corylifol (0, 1, or 10 μg/ml) for 24 h were evaluated and analysed. The data are presented as the mean ± SE of three similar experiments. **F** The correlation coefficients (r) between Corylifol concentration added to chorionic villi cultures and the expression of IDO1, SHP-1, SHP-2, STAT3, pSTAT3 and SOCS3 were − 0.83, − 0.92, − 0.9504, − 0.9248, − 0.87, and 0.732, respectively; the *P* values for the correlations of the levels of IL-6 and IDO1, SHP-1, SHP-2, STAT3, pSTAT3 and SOCS3 were 0.04, 0.03, 0.014, 0.017, 0.03 and 0.042, respectively. **G** The correlation coefficients (r) of the protein expression levels of IDO1 and SHP-1, SHP-2, STAT3, pSTAT3 and SOCS3 were 0.9724, 0.8361, 0.8735, 0.9672 and − 0.9724, respectively; the *P* values for correlations of the protein expression levels of IDO1 and SHP-1, SHP-2, STAT3, pSTAT3 and SOCS3 were 0.010, 0.02, 0.023, 0.004 and 0.0106, respectively. **H** The correlation of the protein expression level of SOCS3 and SHP-1/2 was − 0.8368/− 0.8614, respectively; the *P* value for the correlation of the protein expression levels SOCS3 and SHP-1/2 was 0.026/0.024, respectively The greyscale ratios of IDO1, SOCS3, SHP-1, SHP-2, STAT3 and pSTAT3 and the expression levels of IDO1, SOCS3, SHP-1/2, STAT3 and pSTAT3 were assessed based on the ratios of the greyscale values of the IDO1, SOCS3, SHP-1, SHP-2, STAT3 and pSTAT3 bands to that of the GAPDH band. IDO1, indoleamine 2,3-dioxygenase; SOCS3, suppressors of cytokine signalling 3; STAT3, signal transducer and activator of transcription 3; pSTAT3, phosphorylated STAT3; SHP-1/2, protein tyrosine phosphatase-1/2; IL-6, interleukin-6; GAPDH, glyceraldehyde 3-phosphate dehydrogenase

To further confirm whether IL-6 induces IDO1 expression and regulates SOCS3 expression, tissue explants from chorionic villi and decidua were incubated in medium supplemented with Corylifol A (0.1, 1, or 10 μg/ml) and medium supplemented with IL-6 (2 ng/ml) for 24 h. Then, the explants were lysed for Western blotting analysis. It was found that increasing Corylifol A concentrations in the culture medium decreased the expression of IDO1, SHP-1, SHP-2, STAT3 and pSTAT3 and increased SOCS3 expression (Fig. 7A-B; Fig. 7E-F) in a dose-dependent manner; IDO1 expression was positively correlated with SHP-1, SHP-2, STAT3, and pSTAT3 expression (Fig. 7A and C; Fig. 7E and F), while SOCS3 expression was negatively correlated with SHP-1, SHP-2, STAT3, and pSTAT3 expression (Fig. 7A, B, C, E, F, G) in the tissue explants of chorionic villi and decidua (Fig. 7) cultured in medium containing IL-6 and different concentrations of Corylifol A. The results showed that Corylifol A can decrease the IL-6-dependent upregulation of IDO1 by decreasing the expression of SHP-1, SHP-2, STAT3, and pSTAT3 and by increasing SOCS3 expression.

## Discussion

IDO1 is one of the major proteins that maintain immune tolerance during the first trimester of pregnancy. It has previously been reported that oestrogen upregulates IDO1 expression by downregulating SOCS3 [11] and upregulating transforming growth factor β (TGF-β) [13] expression in the chorionic villi and decidua of women in early pregnancy, thus inducing maternal-foetal tolerance. However, little is known about how IDO1 expression is regulated at the maternal-foetal interface.

### IL-6 upregulates the expression of IDO1 by enhancing SHP-1/2 expression via STAT3 and pSTAT3

Previous studies have shown that IL-6 may reduce IDO1 expression by half by upregulating SOCS3 (but not SHPs) in DCs [14, 15]. However, recent articles have shown that siRNA-mediated inhibition of IL-6 expression reduced the mRNA and protein expression of IDO1 as well as the formation of kynurenines in SKOV-3 and NCI-H596 cells [16] and that IDO1 expression was significantly upregulated in normal human dendritic cells cultured in medium supplemented with IL-6 (50 ng/ml) [17]. Our recent study showed that IL-6 may upregulate the expression of IDO1 [10], and we tried to explore the underlying mechanism in the chorionic villi and decidua of women in early pregnancy. Our results demonstrated that IL-6 expression was positively related to IDO1, SHP-1, SHP-2, STAT3, and pSTAT3 expression in chorionic villus and decidual tissues, which suggested that IL-6 may upregulate the expression of IDO1, SHP-1, SHP-2, STAT3, and pSTAT3 in these tissues. Indeed, according to the Western blotting results, IDO1, SHP-1, SHP-2, STAT3, and pSTAT3 were upregulated in cultured chorionic villus and decidual tissues treated with different concentrations of IL-6 (0 to 100 ng/ml); this IL-6-induced upregulation of IDO1, SHP-1/2, STAT3, and pSTAT3 was blocked by the STAT3 inhibitor Corylifol A, which further confirmed that IL-6 upregulated these proteins. IL-6 has two receptors: one is the cognate IL-6 receptor (IL-6R), and the other is the trans-signal subunit glycoprotein (gp)130 [18, 19]. In the classical IL-6 signalling pathway, IL-6 binds to IL-6R and forms a ligand-receptor complex (IL-6/IL-6R), which then induces receptor dimerization and Janus kinase (JAK)-mediated phosphorylation of the signal transducing subunit gp130. Phosphorylated JAK further induces STAT3 phosphorylation, and STAT3 and pSTAT3 recruit SHP-1/2 and increase the expression of SHP1/2 [19]. IDO1 contains two functional immunoreceptor tyrosine-based inhibitory motifs (ITIMs), which, once phosphorylated at tyrosine residues, can bind to the Src homology 2 (SH2) domain of SHP-1/2 tyrosine phosphatase and form IDO1/SHPs complexes; IDO1/SHPs complexes upregulate IDO1 expression by activating the noncanonical NF-κB pathway and inducing the production of type I IFNs (IFNα/β) and TGF-β [14, 15]. Therefore, our results revealed that IL-6 upregulated IDO1 expression in part by enhancing SHP-1/2 expression via STAT3 and pSTAT3 in the chorionic villi and decidua of women in early pregnancy.

### IL-6 regulates the expression of SOCS3

SOCS3, which is a member of a protein family that binds to cytokine receptors, negatively regulates various cytokines by suppressing cytokine signalling. SOCS3 is an essential regulator of the leukaemia inhibitory factor receptor signalling pathway that contributes to trophoblast differentiation [20]. SOCS3 expression is decreased in the placentas of women with intrahepatic cholestasis [21], which suggests an essential role of SOCS3 in the pathogenesis of this disease. Downregulated SOCS3 in trophoblasts may lead to weaker inhibitory effects of cytokines on the placentas of patients with preeclampsia, which may account for the increased placental inflammatory response in preeclampsia [22].

IL-6 is a key regulator of SOCS-3 induction that ats by binding to its IL-6 receptors on the cell membrane. In the classical IL-6 signalling pathway, IL-6 phosphorylates STAT3, and phosphorylated STAT3 translocates to the nucleus, where it recognizes specific elements in the promoter of the SOCS-3 gene and induces SOCS-3 expression [19, 23, 24]. However, recent studies have demonstrated that the relationship among IL-6, STAT3, and SOCS3 is more complicated; these reports showed that IL-6 could promote SOCS3 expression in mouse spermatogonial cells (GC-1 spg cells) treated with IL-6 (10, or 50, or 100 ng/ml) for 24 h; this upregulation of SOCS3 was associated with the decreased expression of phosphorylated STAT3 but was not associated with the expression of STAT3 [25]. Furthermore, IL-6 could reduce SOCS3 expression in human mast cell and pancreatic cancer cell lines treated with IL-6 (100 ng/ml) for 24 h and in colorectal tissues from ulcerative colitis carcinogenesis animal models (ICR male mice); this downregulation of SOCS3 was associated with increased methylation of SOCS3 by DNA methyltransferase 1, increased STAT3 expression and increased STAT3 phosphorylation [26–29].

Our results demonstrated that IL-6 expression was negatively related to SOCS3 and positively related to IDO1, SHP-1, SHP-2, STAT3, and pSTAT3 in chorionic villus and decidual tissues, which suggested the possibility that IL-6 may simultaneously upregulate the expression of IDO1, SHP-1/2, STAT3, and pSTAT3 and downregulate SOCS3 expression in tissues. However, as shown by the Western blotting and qRT–PCR results, a low concentration of IL-6 (0–2 ng/ml) may upregulate SOCS3 expression in a dose-dependent manner, while a high concentration of IL-6 (2–100 ng/ml) may decrease SOCS3 expression in a dose-dependent manner, and SOCS3 expression was decreased to normal levels in the cultured chorionic villus and decidual tissues when the IL-6 concentration reached 100 ng/ml. This IL-6 mediated increase or decrease in SOCS3 expression can be blocked by the STAT3 inhibitor Corylifol A, which further confirmed that IL-6 regulated protein expression.

According to the classical IL-6 signalling pathway, IL-6 activates STAT3; activated STAT3 then drives biological responses, one of which is the upregulation of IDO1, which simultaneously induces SOCS-3 expression. Then, SOCS3 negatively regulates IL-6 signalling and terminates the JAK/STAT signalling cascade, forming a negative feedback loop that allows the cell to return to its basal state [19, 24]. Nevertheless, how IL-6 and activated STAT3 induce SOCS-3 expression is not well understood.

Our data indicated that whether IL-6 and STAT3 upregulate or downregulate SOCS3 expression is dependent on the concentration of IL-6 that was added to culture medium of the chorionic villi and decidual tissues; a low concentration of IL-6 (0–2 ng/ml) positively regulated SOCS3 expression by increasing and activating STAT3 to lower degrees, while a high concentration of IL-6 (2–100 ng/ml) negatively regulated SOCS3 expression by increasing and activating STAT3 to higher degrees. This is partly different from previous reports that demonstrate that regulation of SOCS3 was associated with the increased or decreased expression of STAT3 and phosphorylated STAT3 [24–29].

### IL-6 regulates IDO1 expression by regulating SOCS3 expression

IDO1 contains two functional ITIMs, which, once tyrosine phosphorylated, can bind to either SOCS3 or SHP-1/2. SOCS3 binds to IDO1 via its Src homology-2 (SH2) domain, thus interacting with the immunoreceptor tyrosine-based inhibitory motifs of the enzyme, forming SOCS3/SHPs complexes, and resulting in the formation of an E3 ubiquitin ligase complex that drives the proteasomal degradation of SOCS3-bound IDO1 [30, 31]. SOCS3 downregulates IDO1 expression in chorionic villi and decidua, which suggests that SOCS3 plays an important role in maintaining the balance necessary for immunotolerance [11]. SHP-1/2 binds to phosphorylated ITIMs of IDO1 with its SH2 domain and forms IDO1/SHPs complexes; the IDO1/SHPs complexes activate the noncanonical NF-κB pathway (p52/RelB) via the inhibition of IRAK1, which in turn induces the production of type I IFNs (IFNα/β) and TGF-β, which synergize with noncanonical NF-κB signalling to upregulate IDO1 expression [14, 15].

It has been reported that whether the ITIMs of IDO1 bind to SOCS3 or SHP-1/2 in plasmacytoid DCs (pDCs) depends on exposure to environmental stimuli. In a TGF-β-dominated microenvironment, TGF-β phosphorylates the ITIM domains in IDO1 by activating Fyn (a tyrosine kinase that belongs to the Src family). The phosphorylated ITIMs of IDO1 bind to SHP-1/2 but not to SOCS3 and form IDO1/SHPs complexes, resulting in IDO1 upregulation. On the other hand, in an IL-6-dominated microenvironment, IL-6 upregulates SOCS3 and promotes the binding of SOCS3 to the ITIMs of IDO1 (which are phosphorylated under these conditions by unknown mechanisms), thus contributing to the formation of SOCS3/SHPs complexes and resulting in the degradation of IDO1 [14, 15].

However, our data indicated that in an IL-6-dominated microenvironment, IL-6 upregulates the expression of both IDO1 and SHP-1/2, which suggested the possibility that SHP-1/2 can bind to IDO1, forming IDO1/SHPs complexes, and that these complexes can upregulate IDO1 expression; this might be different from previous studies, in which IDO1 could not bind to SHP-1/2 in response to IL-6 stimulation [14, 15]. Further study is needed to confirm the possibility that SHP-1/2 can bind to IDO1 in response to IL-6 stimulation. Our data indicated that in a microenvironment with low concentrations of IL-6 (0–2 ng/ml), SOCS3 does not downregulate IDO1 expression in chorionic villi and decidua because SOCS3 expression was positively related to IDO1, SHP-1/2, STAT3, and pSTAT3 expression; IL-6 was positively related to the expression of SOCS3 in response to stimulation with low concentrations of IL-6, suggesting the possibility that SOCS3 cannot bind to IDO1 and form IDO1/SOCS3 complexes in a microenvironment with low concentrations of IL-6; this might be different from previous reports, in which SOCS3 could bind to IDO1 and degrade IDO1 in response to IL-6 stimulation [14, 15]. Our data indicated that in a microenvironment with high concentrations of IL-6 (2–100 ng/ml), SOCS3 downregulated IDO1 expression because SOCS3 expression was negatively related to IDO1, SHP-1/2, STAT3, and pSTAT3 expression and negatively related to the concentration of IL-6 added to the chorionic villi and decidua cultures, suggesting the possibility that SOCS3 can bind to IDO1, form IDO1/SOCS3 complexes, and degrade IDO1 in response to high concentrations of IL-6; however, this ability of SOCS3 to downregulate IDO1 was gradually weakened by the addition of increasing concentrations of IL-6, because SOCS3 expression was negatively related to the concentration of IL-6 in chorionic villi and decidua cultures with high concentrations of IL-6, this may be partly different from reports that showed that SOCS3 can bind to IDO1 and degrade IDO1 in response to IL-6 stimulation [14, 15].

### IL-6 positively regulates the expression of IDO1 and negatively regulates the expression of SOCS3 in the chorionic villi and decidua of women in early pregnancy

Our results demonstrated that IL-6 expression was negatively related to SOCS3 expression, and the expression of IL-6 and SOCS3 was positively and negatively related to IDO1, SHP-1, SHP-2, STAT3, and pSTAT3, respectively, in chorionic villus and decidual tissues and in chorionic villi and decidua cultured with high concentrations of IL-6 (2–100 ng/ml); these results suggested that IL-6 exerted similar effects on IDO1 and SOCS3 in tissues in vivo and in tissues cultured with high concentrations of IL-6 (2–100 ng/ml). The IL-6 levels from the cultured explants of human placentas treated with progesterone are approximately 10–20 ng/g (15.4 ± 4.1 ng/g) of tissue, which is similar to the baseline content of IL-6 in the placenta [32]. In our experiments, 0.5 g explants of chorionic villus and decidual tissues were cultured in 1 ml medium that contained IL-6 at concentrations of 0, 0.5, 2, 10, 50, or 100 ng/ml, which are equal to 0, 1, 4, 20, 100, and 200 ng/g of tissues. Our results indicated that the baseline content of IL-6 (10–20 ng/g or 5–10 ng/ml) upregulates IDO1 expression and downregulates SOCS3 expression. Therefore, in normal chorionic villi and decidua of women in early pregnancy, IL-6 upregulates IDO1 expression in a dose-dependent manner partly by enhancing SHP-1/2 expression via STAT3 and pSTAT3. Additionally, IL-6 downregulates SOCS3 expression, and SOCS3 downregulates IDO1 expression in a dose-dependent manner, suggesting that a high expression of IL-6 may lead to greater IDO1 expression, lower SOCS-3 expression, and reduced degradation of IDO1 by SOCS3. It has been reported that pathological concentrations of IL-6 (16 ng/ml) do not cause amnion cell ageing, cell death, cellular transitions, or inflammation and that IL-6 may function to maintain cellular homeostasis of foetal membrane cells throughout gestation [33]. Our data indicate that high expression of IL-6 may be better for maintaining immunological tolerance at the maternal-foetal interface.

## Conclusion

**Fig. 8:**
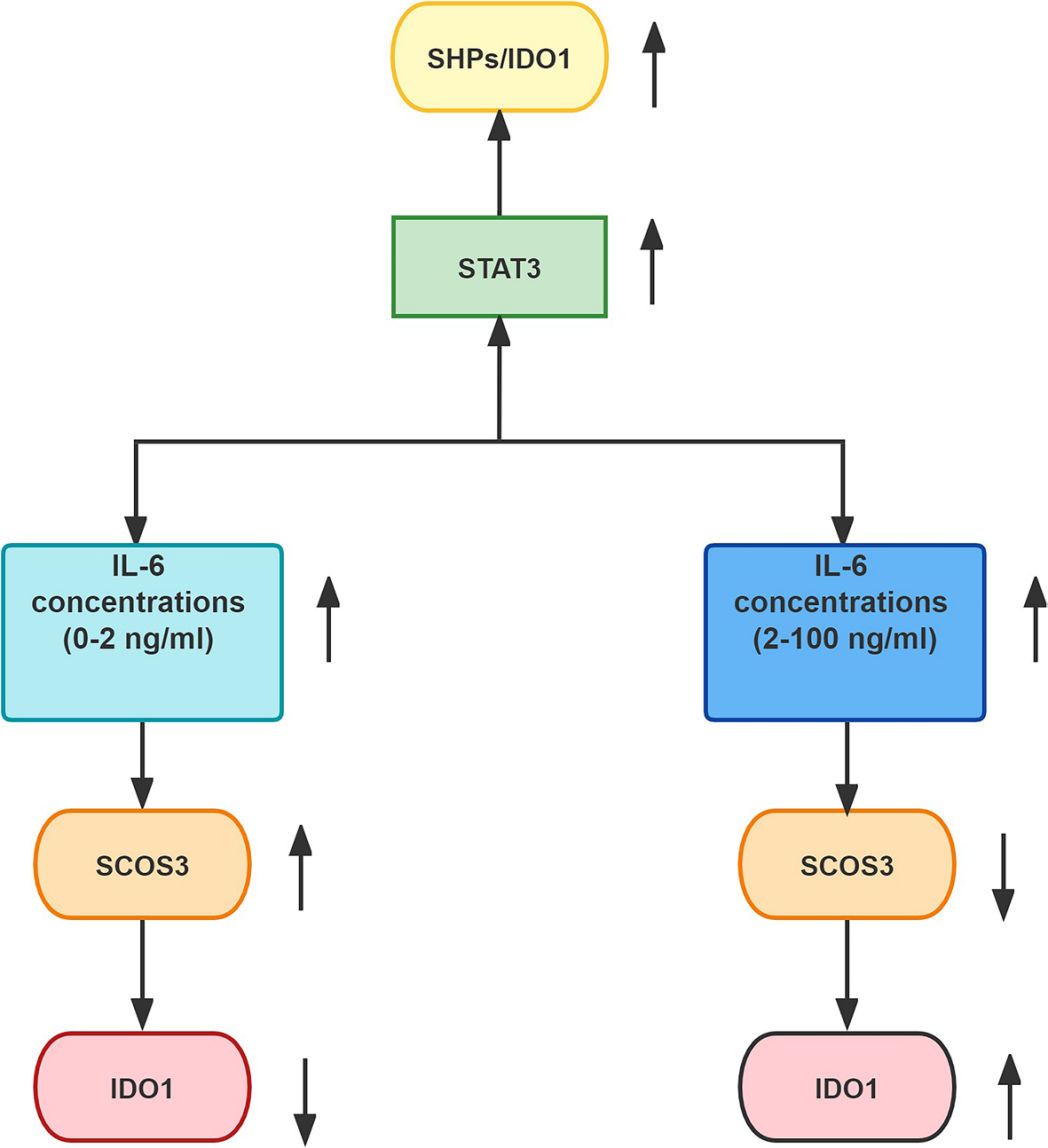
Diagram illustrating the molecular mechanism involved in the induction of IDO1 by IL-6

In conclusion, in the normal physiological state of pregnancy, IL-6, STAT3, pSTAT3, SHP1/2, SOCS3 and IDO1 are expressed in chorionic villus and decidual tissues during early pregnancy, IL-6 upregulates the expression of IDO1, STAT3, pSTAT3 and SHP1/2 in a dose-dependent manner, and IL-6 upregulates the expression of IDO1 by enhancing SHP-1/2 expression via STAT3 and pSTAT3. IL-6 upregulates the expression of IDO1 and simultaneously negatively regulates the expression of SOCS3. High expression of IDO1 may be maintained by increasing the physiological expression of IL-6, which induces higher IDO1 expression, lower SOCS-3 expression, and reduced degradation of IDO1. High expression of IL-6 may be better for maintaining immunological tolerance at the maternal-foetal interface. A diagram illustrating the molecular mechanism involved in the induction of IDO1 by IL-6 is provided as follows (Fig. 8):

## Supplementary Information

**Additional file 1.** Supplementary Table

**Additional file 2.** Supplementary Figure

## Data Availability

All data generated or analysed during this study are included in this published article and its supplementary information files.

## Abbreviations

TCVD: Tissues of chorionic villi and decidua
IDO1: Indoleamine 2,3-dioxygenase1
Th: T helper
PVDF: Polyvinylidene fluoride
GAPDH: Glyceraldehyde-3-phosphate dehydrogenase
HRP: Horseradish peroxidase
qRT–PCR: Quantitative reverse transcription-polymerase chain reaction
TGF-β: Transforming growth factor beta
SDS–PAGE SDS: Polyacrylamide gel electrophoresis
DAB: Diaminobenzidine
TGF-β: Transforming growth factor beta
gp: Glycoprotein
IL-6R: IL-6 receptor
ITIM: Inhibitory motifs
