# Supplementary material for: Molecular mechanisms involved in the IL-6-mediated upregulation of indoleamine 2,3-dioxygenase 1 (IDO1) expression in the chorionic villi and decidua of women in early pregnancy
Source: BMC Pregnancy Childbirth. 2022 Dec 31;22:983. doi: 10.1186/s12884-022-05307-5 (PMC9805015; doi:10.1186/s12884-022-05307-5)
Supplement: Supplementary file 1 — Additional file 1. Supplementary Table [file 12884_2022_5307_MOESM1_ESM.docx]

**Supplementary Table 1.** IDO copy number in cultured chorionic villi added with IL-6(analyzed by qRT-PCR)

| IL-6 concentration in cultured medium of villi(ng/ml) | GAPDH Ct (Mean±SD) | IDO Ct (Mean±SD) | IDO(2^-ΔΔct^)(Mean ±SD) |
| --- | --- | --- | --- |
| 0(control) | 16.69±0.13 | 24.00±0.14 | 1* |
| 0.5 | 16.37±0.22 | 23.38±0.13 | 1.23± 0.03** |
| 2 | 16.69±0.15 | 23.48±0.25 | 1.43 ±0.02*** |
| 10 | 16.69±0.15 | 23.38±0.34 | 1.64 ±0.03**** |
| 50 | 16.44±0.11 | 22.76±0.11 | 1.99 ±0.01***** |
| 100 | 16.27±0.25 | 22.48±0.13 | 2.14 ±0.02 |

Note: Each experiment is performed in duplicate. **P*=0.0375,0.0106, 0.0003,<0.0001,<0.0001, compared with the groups treated with 0.5 ng/ml, 2 ng/ml,10 ng/ml,50 ng/ml and 100 ng/ml of IL-6, respectively; ***P*=0.021, 0.0014, 0.0007, <0.0001, compared with the groups treated with 2 ng/ml, 10 ng/ml, 50 ng/ml and 100 ng/ml of IL-6, respectively; ****P*= 0.037, 0.0021, <0.0001, compared with the groups treated with 10 ng/ml, 50 ng/ml and 100 ng/ml of IL-6, respectively; *****P*=0.001 and 0.00041, compared with the groups treated with 50 ng/ml and 100 ng/ml of IL-6, respectively; ******P*= 0.038, compared with the groups treated with 100 ng/ml of IL-6. Control: cultured tissues of chorionic villi without IL-6; IDO, indoleamine 2,3-dioxygenase; GAPDH, glyceraldehyde 3-phosphate dehydrogenase; qRT-PCR, Quantitative real time polymerase chain reaction.

**Supplementary Table 2.** IDO copy number in cultured decidua added with IL-6(analyzed by qRT-PCR)

| IL-6 concentration in cultured medium of decidua(ng/ml) | GAPDH Ct (Mean±SD) | IDO Ct (Mean±SD) | IDO(2^-ΔΔct^)(Mean ±SD) |
| --- | --- | --- | --- |
| 0(control) | 15.36±0.21 | 22.81±0.09 | 1* |
| 0.5 | 15.22±0.13 | 22.38±0.04 | 1.22±0.03** |
| 2 | 15.13±0.27 | 22.08±0.07 | 1.41±0.04*** |
| 10 | 15.06±0.14 | 21.68±0.15 | 1.78±0.05**** |
| 50 | 15.77±0.16 | 22.26±0.01 | 1.95±0.01***** |
| 100 | 15.88±0.14 | 22.18±0.02 | 2.22±0.02 |

Note: Each experiment is performed in duplicate. **P*= 0.025, 0.0206, 0.0003, <0.0001, <0.0001, compared with the groups treated with 0.5ng/ml, 2 ng/ml,10 ng/ml,50 ng/ml and 100 ng/ml of IL-6, respectively; ***P*=0.031, 0.0027, 0.0006, <0.0001, compared with the groups treated with 2 ng/ml,10 ng/ml,50 ng/ml and 100 ng/ml of IL-6, respectively; ****P*=0.032, 0.00273, <0.0001, compared with the groups treated with 10 ng/ml, 50 ng/ml and 100 ng/ml of IL-6, respectively; *****P*=0.002 and 0.0011, compared with the groups treated with 50 ng/ml and 100 ng/ml of IL-6, respectively; ******P*= 0.031, compared with the groups treated with 100 ng/ml of IL-6. Control: cultured tissues of chorionic villi without IL-6; IDO, indoleamine 2,3-dioxygenase; GAPDH, glyceraldehyde 3-phosphate dehydrogenase; qRT-PCR, Quantitative real time polymerase chain reaction.

**Supplementary Table 3.** SOCS3 copy number in cultured chorionic villi added with IL-6 (analyzed by qRT-PCR)

| IL-6 concentration in cultured medium of decidua(ng/ml) | GAPDH Ct (Mean±SD) | SOCS3 Ct (Mean±SD) | SOCS3(2^-ΔΔct^)(Mean ±SD) |
| --- | --- | --- | --- |
| 0(control) | 18.13±0.14 | 22.81±0.12 | 1* |
| 0.5 | 18.71±0.11 | 22.28±0.18 | 1.68 ±0.02** |
| 2 | 18.34±0.23 | 22.48±0.21 | 2.06 ±0.03*** |
| 10 | 18.43±0.27 | 22.55±0.16 | 1.47 ±0.02**** |
| 50 | 18.54±0.18 | 22.95±0.22 | 1.21 ±0.03***** |
| 100 | 18.81±0.17 | 23.48±0.11 | 1.01 ±0.01 |

Note: Each experiment is performed in duplicate. *P= 0.0064, <0.0001, 0.0097, 0.033, >0.05, compared with the groups treated with 0.5ng/ml, 2 ng/ml,10 ng/ml,50 ng/ml and 100 ng/ml of IL-6, respectively; **P=0.063, >0.05, 0.0097, 0.0072, compared with the groups treated with 2 ng/ml,10 ng/ml, 50 ng/ml and 100 ng/ml of IL-6, respectively; ***P=0.0012, 0.0006, <0.0001, compared with the groups treated with 10 ng/ml, 50 ng/ml and 100 ng/ml of IL-6, respectively;****P=0.0014 and 0.007, compared with the groups treated with 50 ng/ml and 100 ng/ml of IL-6, respectively; *****P= 0.039, compared with the groups treated with 100 ng/ml of IL-6. Control: cultured tissues of chorionic villi without IL-6; SOCS3, suppressors of cytokine signaling 3; GAPDH, glyceraldehyde 3-phosphate dehydrogenase; qRT-PCR, Quantitative real time polymerase chain reaction.

**Supplementary Table 4.** SOCS3 copy number in cultured decidua added with IL-6 (analyzed by qRT-PCR)

| IL-6 concentration in cultured medium of decidua(ng/ml) | GAPDH Ct (Mean±SD) | SOCS3 Ct (Mean±SD) | SOCS3(2^-ΔΔct^)(Mean ±SD) |
| --- | --- | --- | --- |
| 0(control) | 19.34±0.22 | 25.04±0.13 | 1* |
| 0.5 | 19.16±0.11 | 24.29±0.22 | 1.48 ±0.02** |
| 2 | 19.44±0.32 | 24.12±0.15 | 2.03 ±0.02*** |
| 10 | 19.32±0.24 | 24.44±0.14 | 1.49 ±0.01**** |
| 50 | 19.22±0.21 | 24.69±0.21 | 1.17 ±0.02***** |
| 100 | 19.65±0.13 | 25.29±0.09 | 1.04 ±0.03 |

Note: Each experiment is performed in duplicate. *P=0.0072, <0.0001, 0.041, 0.025, >0.05, compared with the groups treated with 0.5 ng/ml, 2 ng/ml, 10 ng/ml, 50 ng/ml and 100 ng/ml of IL-6, respectively; **P= 0.023, 0.0017, 0.0073, <0.0001, compared with the groups treated with 2 ng/ml,10 ng/ml,50 ng/ml and 100 ng/ml of IL-6, respectively; ***P=0.0014, 0.0007, <0.0001, compared with the groups treated with 10 ng/ml, 50 ng/ml and 100 ng/ml of IL-6, respectively;**** P=0.0024 and 0.0019, compared with the groups treated with 50 ng/ml and 100 ng/ml of IL-6, respectively; *****P= 0.034, compared with the groups treated with 100 ng/ml of IL-6. Control: cultured tissues of chorionic villi without IL-6; SOCS3, suppressors of cytokine signaling 3; GAPDH, glyceraldehyde 3-phosphate dehydrogenase; qRT-PCR, Quantitative real time polymerase chain reaction.
