# Supplementary material for: Molecular mechanisms involved in the IL-6-mediated upregulation of indoleamine 2,3-dioxygenase 1 (IDO1) expression in the chorionic villi and decidua of women in early pregnancy
Source: BMC Pregnancy Childbirth. 2022 Dec 31;22:983. doi: 10.1186/s12884-022-05307-5 (PMC9805015; doi:10.1186/s12884-022-05307-5)
Supplement: Supplementary file 2 — Additional file 2. Supplementary Figure [file 12884_2022_5307_MOESM2_ESM.pdf]

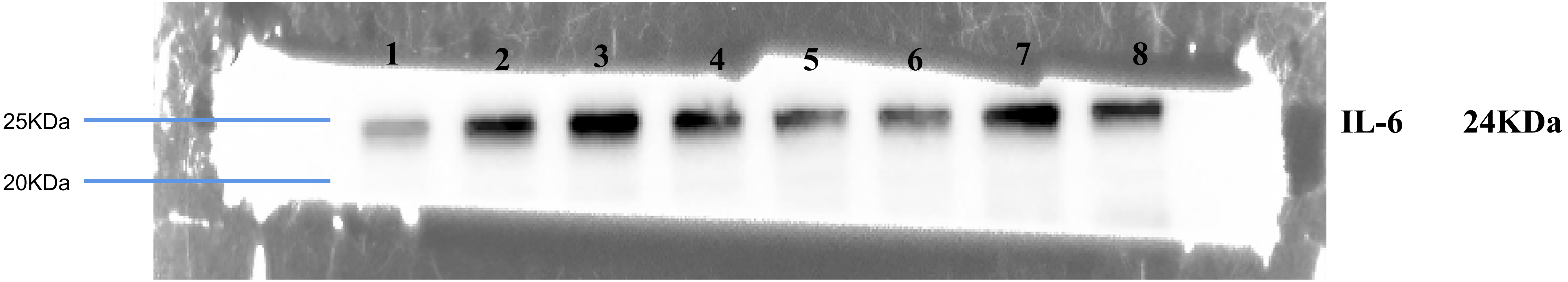

Figure 1A Expression of IL-6 in chorionic villi. Labels 1-8 represent different samples from healthy pregnant women

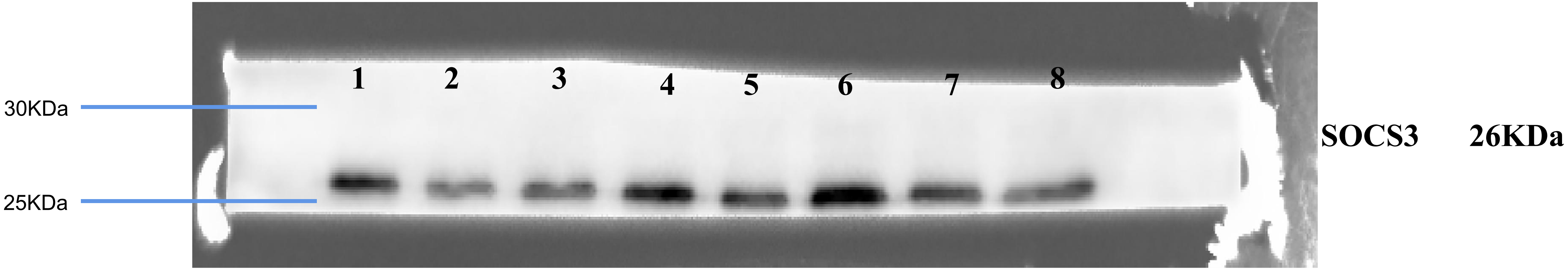

Figure 1A Expression of SOCS3 in chorionic villi. Labels 1-8 represent different samples from healthy pregnant women

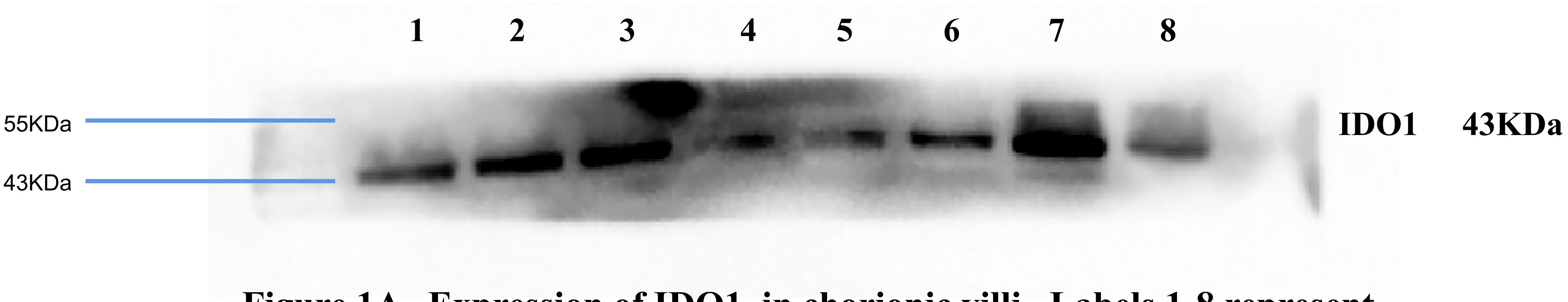

Figure 1A Expression of IDO1 in chorionic villi. Labels 1-8 represent different samples from healthy pregnant women

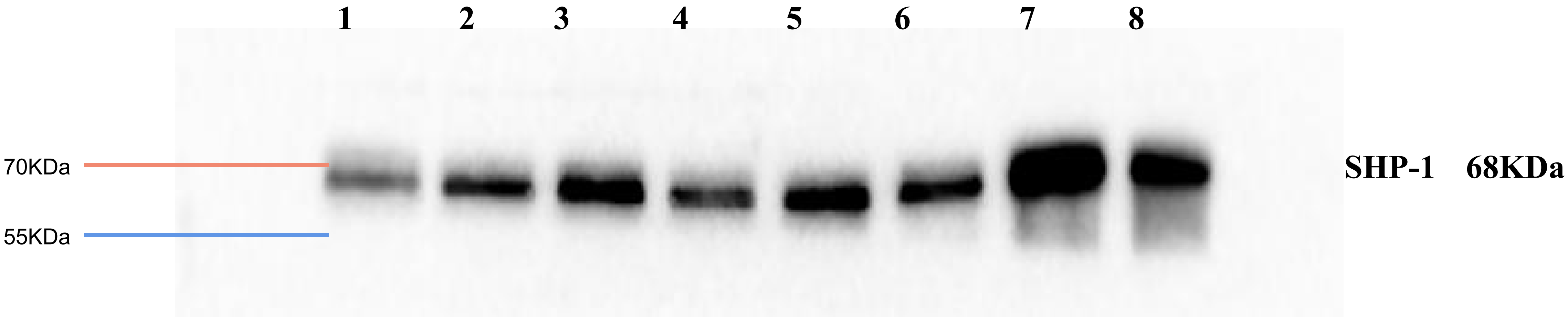

Figure 1A Expression of SHP-1 in chorionic villi. Labels 1-8 represent different samples from healthy pregnant women

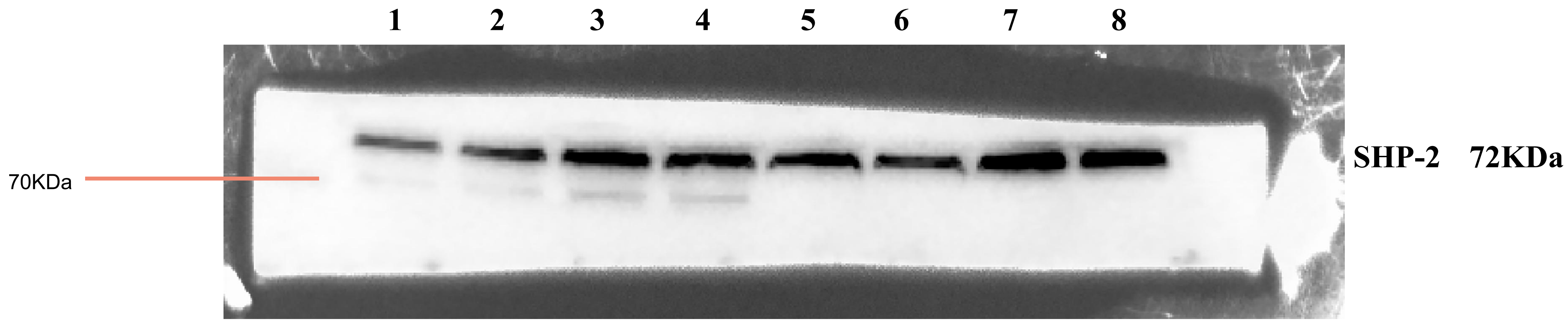

Figure 1A Expression of SHP-2 in chorionic villi. Labels 1-8 represent different samples from healthy pregnant women

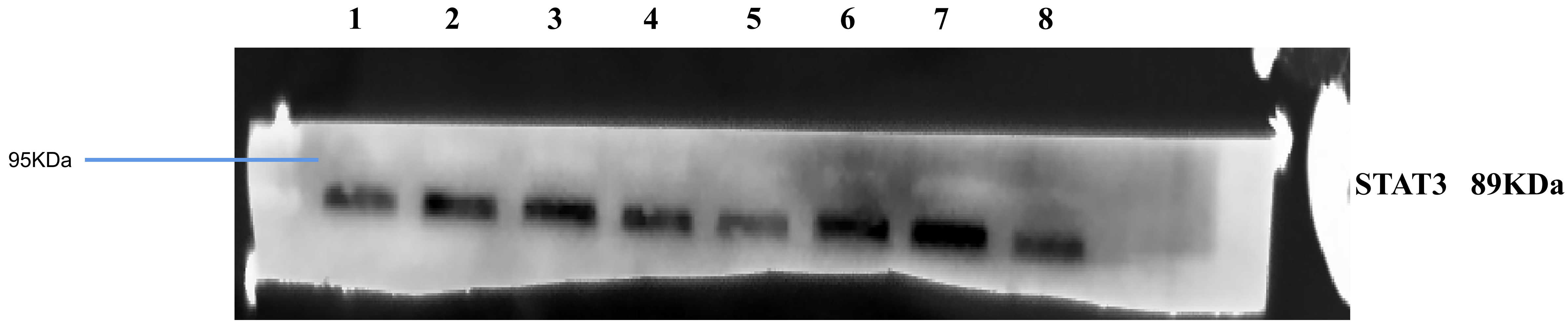

Figure 1A Expression of STAT3 in chorionic villi. Labels 1-8 represent different samples from healthy pregnant women

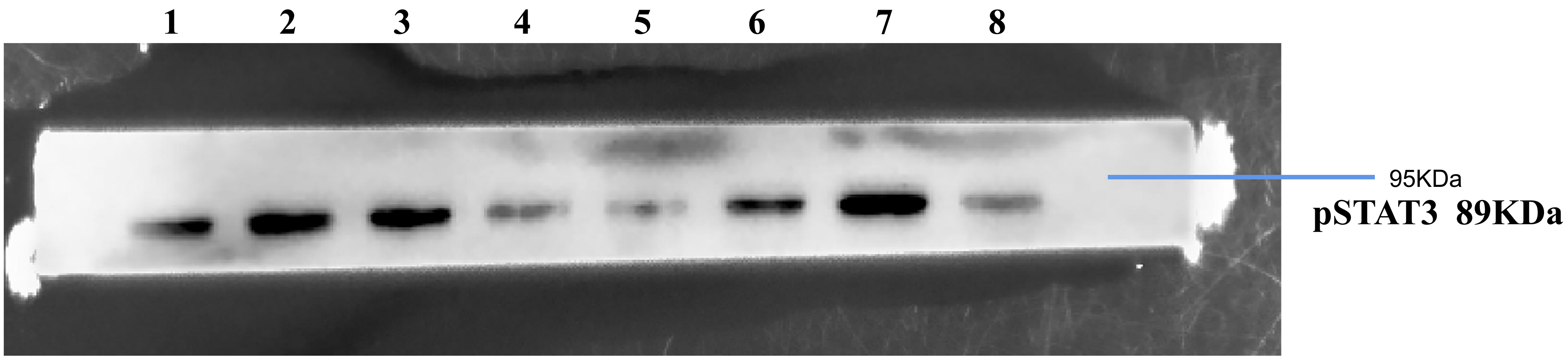

Figure 1A Expression of pSTAT3 in chorionic villi. Labels 1-8 represent different samples from healthy pregnant women

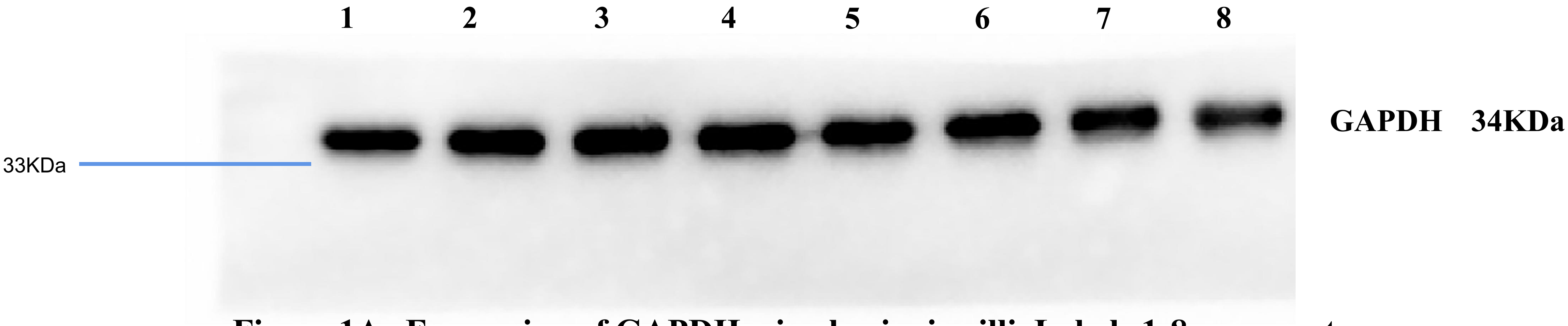

Figure 1A Expression of GAPDH in chorionic villi. Labels 1-8 represent different samples from healthy pregnant women

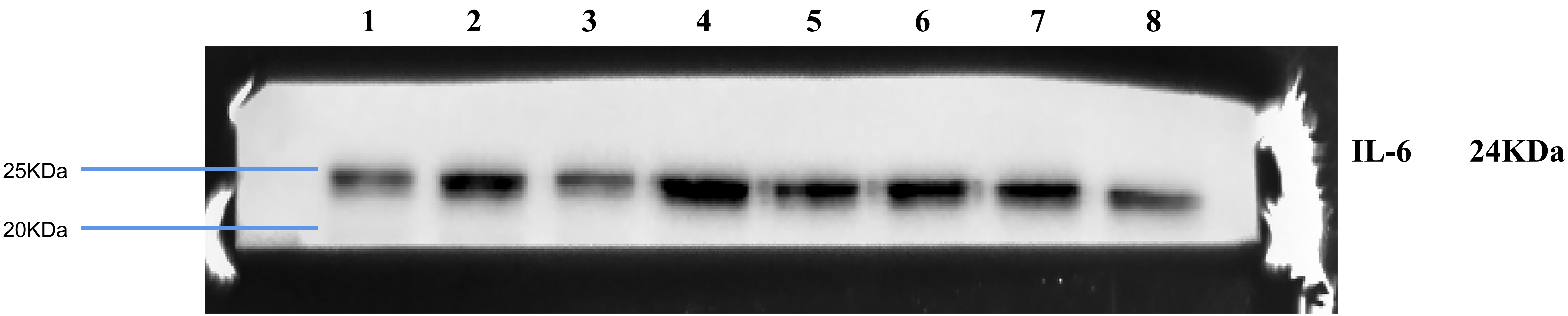

Figure 1E Expression of IL-6 in decidua. Labels 1-8 represent different samples from healthy pregnant women

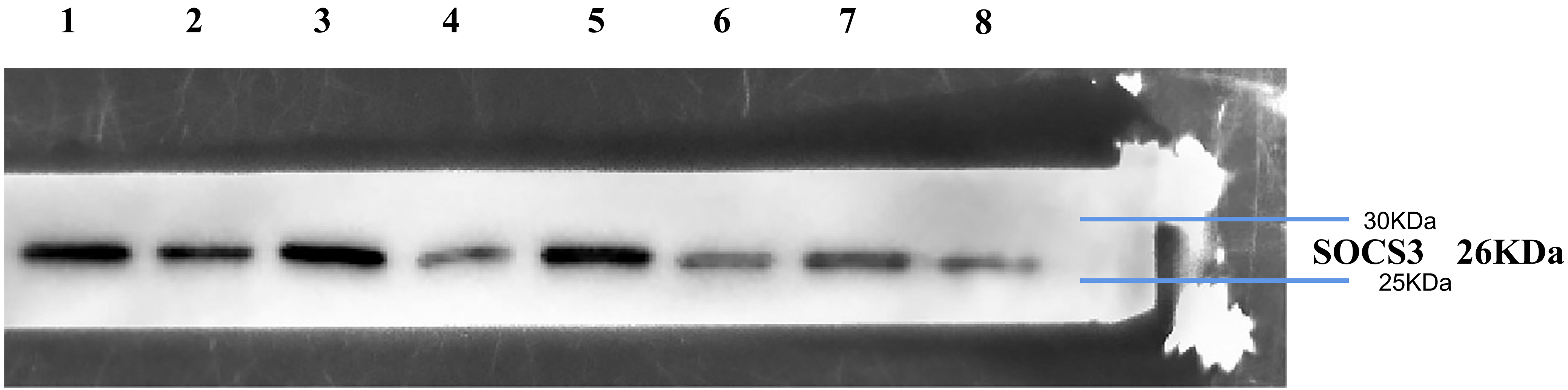

Figure 1E Expression of SOCS3 in decidua. Labels 1-8 represent different samples from healthy pregnant women

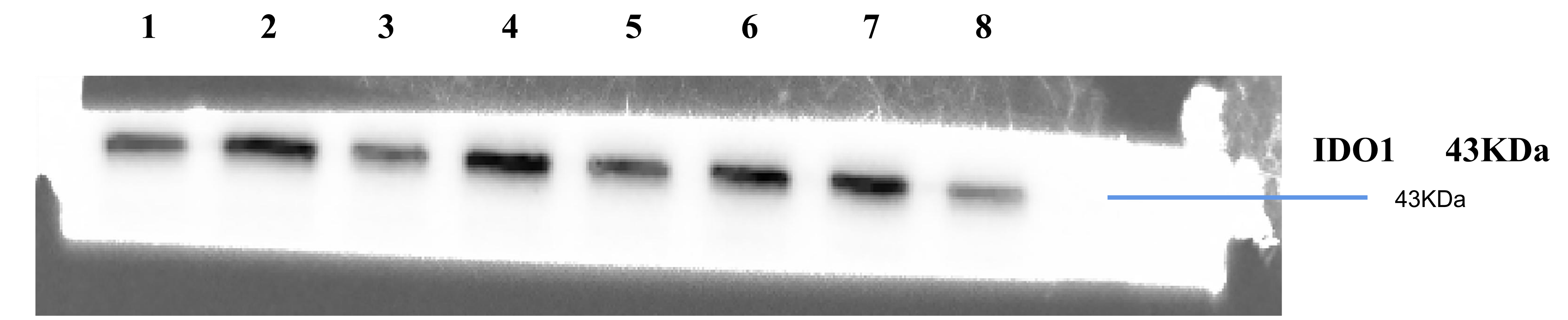

Figure 1E Expression of IDO1 in decidua. Labels 1-8 represent different samples from healthy pregnant women

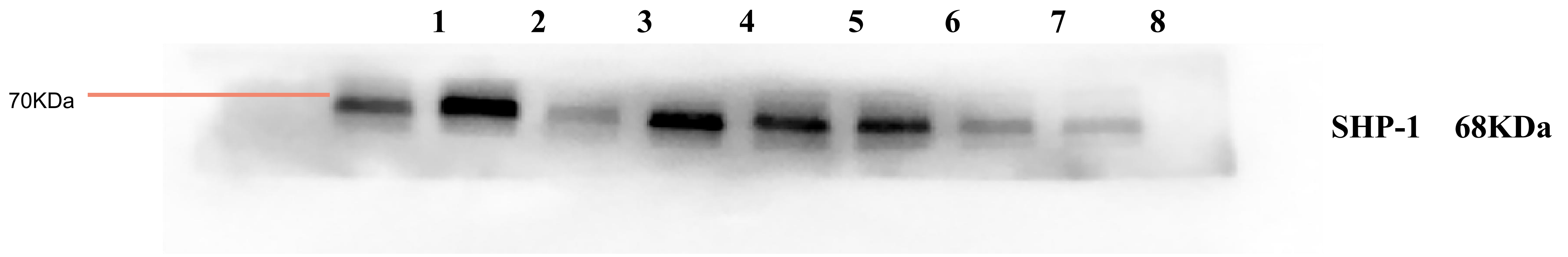

Figure 1E Expression of SHP-1 in decidua. Labels 1-8 represent different samples from healthy pregnant women

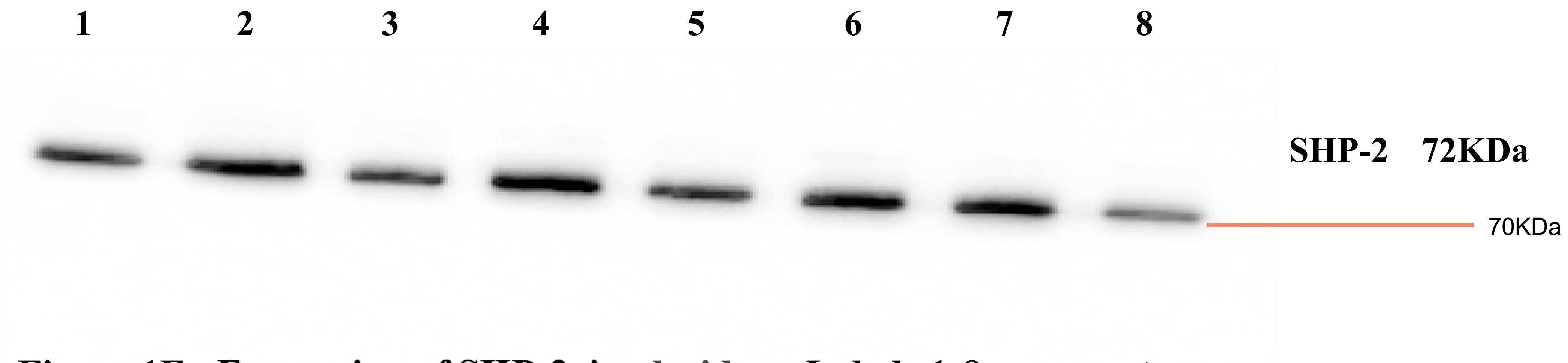

Figure 1E Expression of SHP-2 in decidua. Labels 1-8 represent different samples from healthy pregnant women

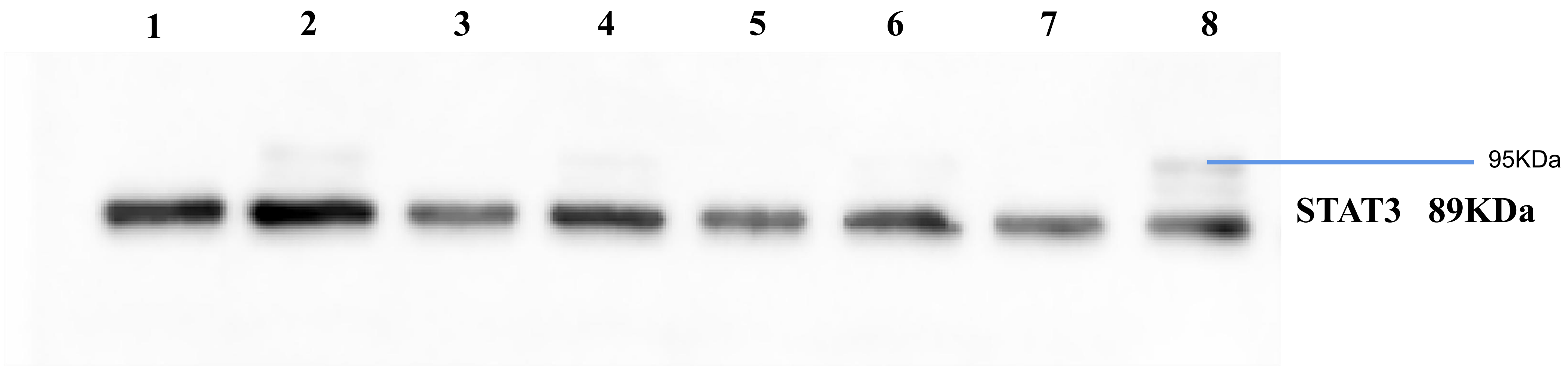

Figure 1E Expression of STAT3 in decidua. Labels 1-8 represent different samples from healthy pregnant women

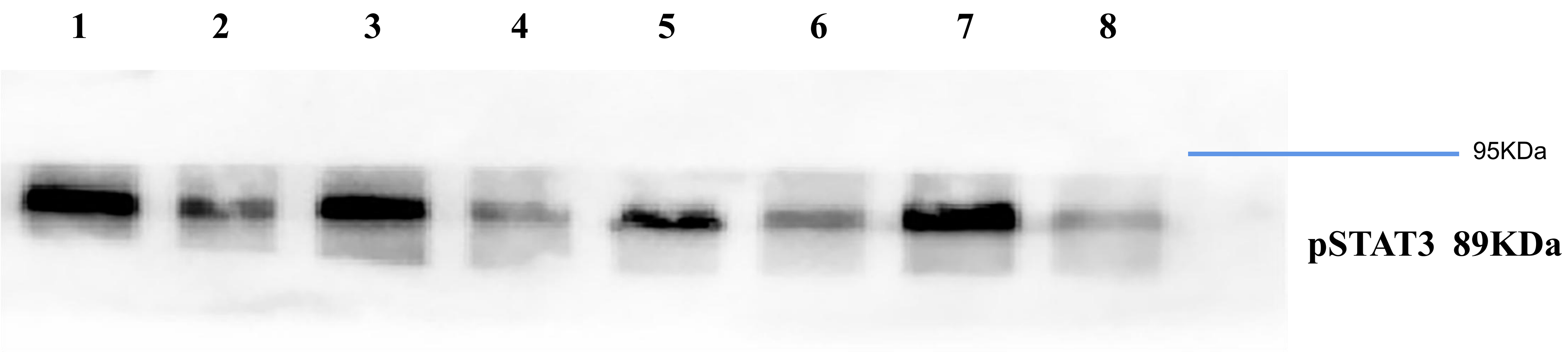

Figure 1E Expression of **pSTAT3** in decidua. Labels 1-8 represent different samples from healthy pregnant women

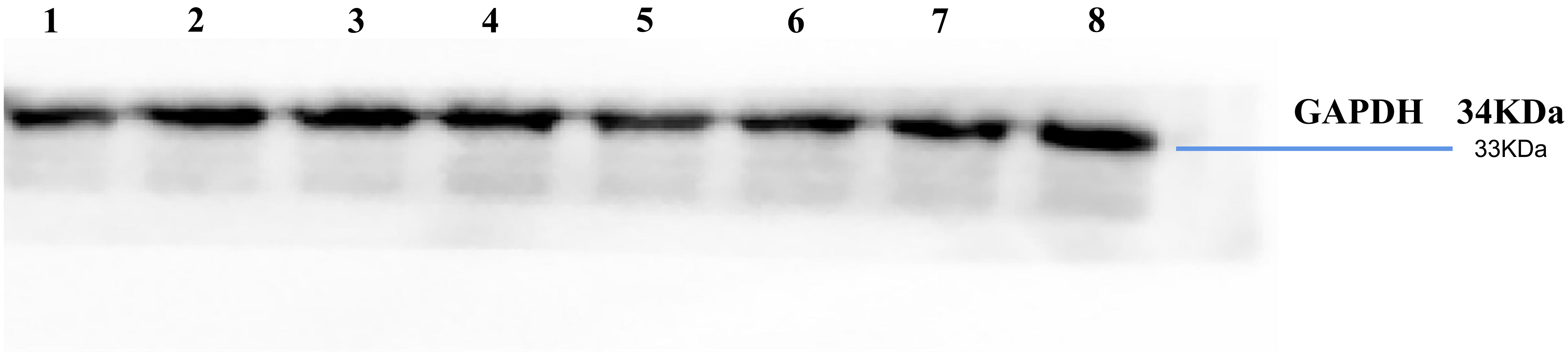

Figure 1E Expression of **GAPDH** in decidua. Labels 1-8 represent different samples from healthy pregnant women

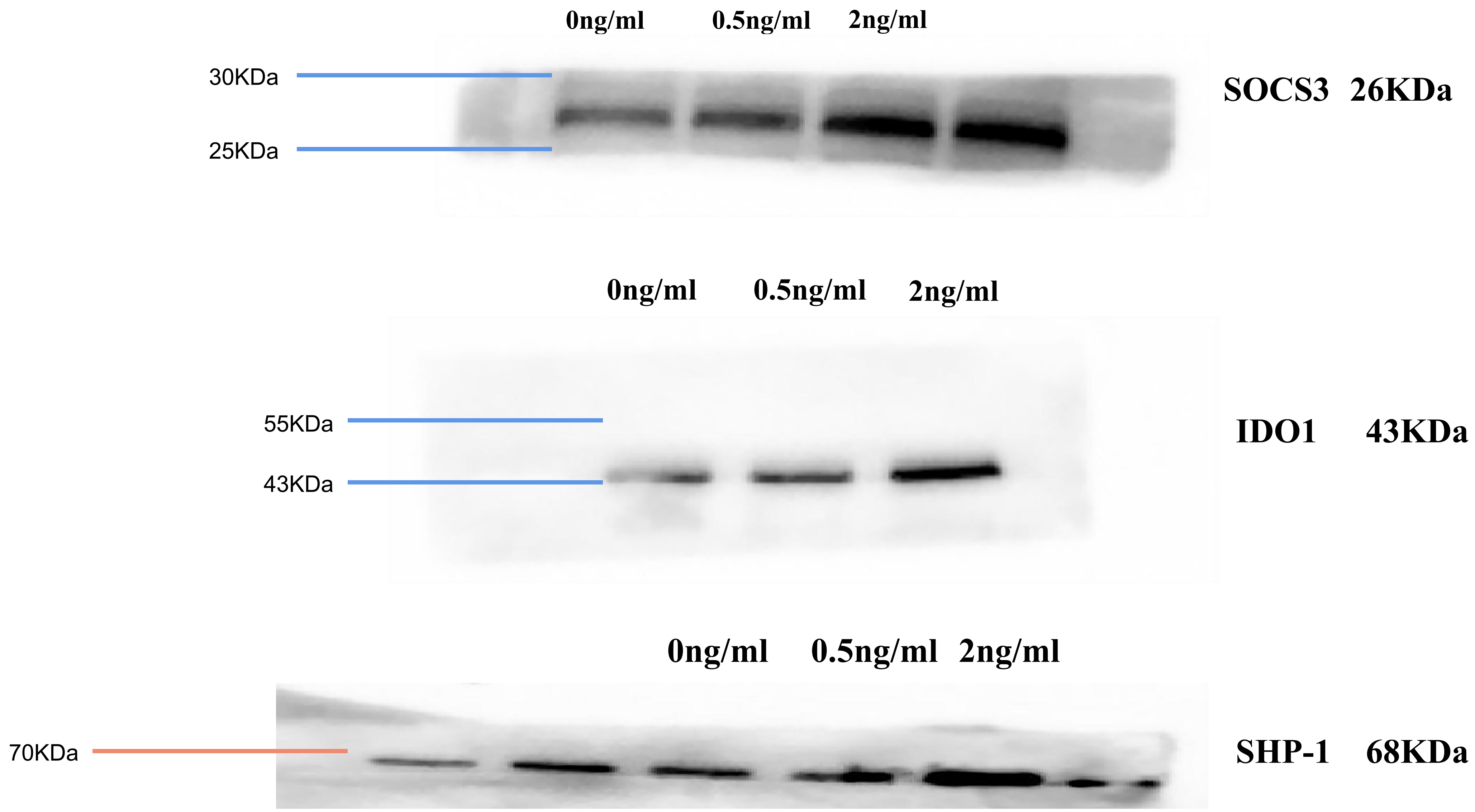

Figure 3A SOCS3,IDO1 and SHP-1 expression in cultured villi added with low concentration of IL-6

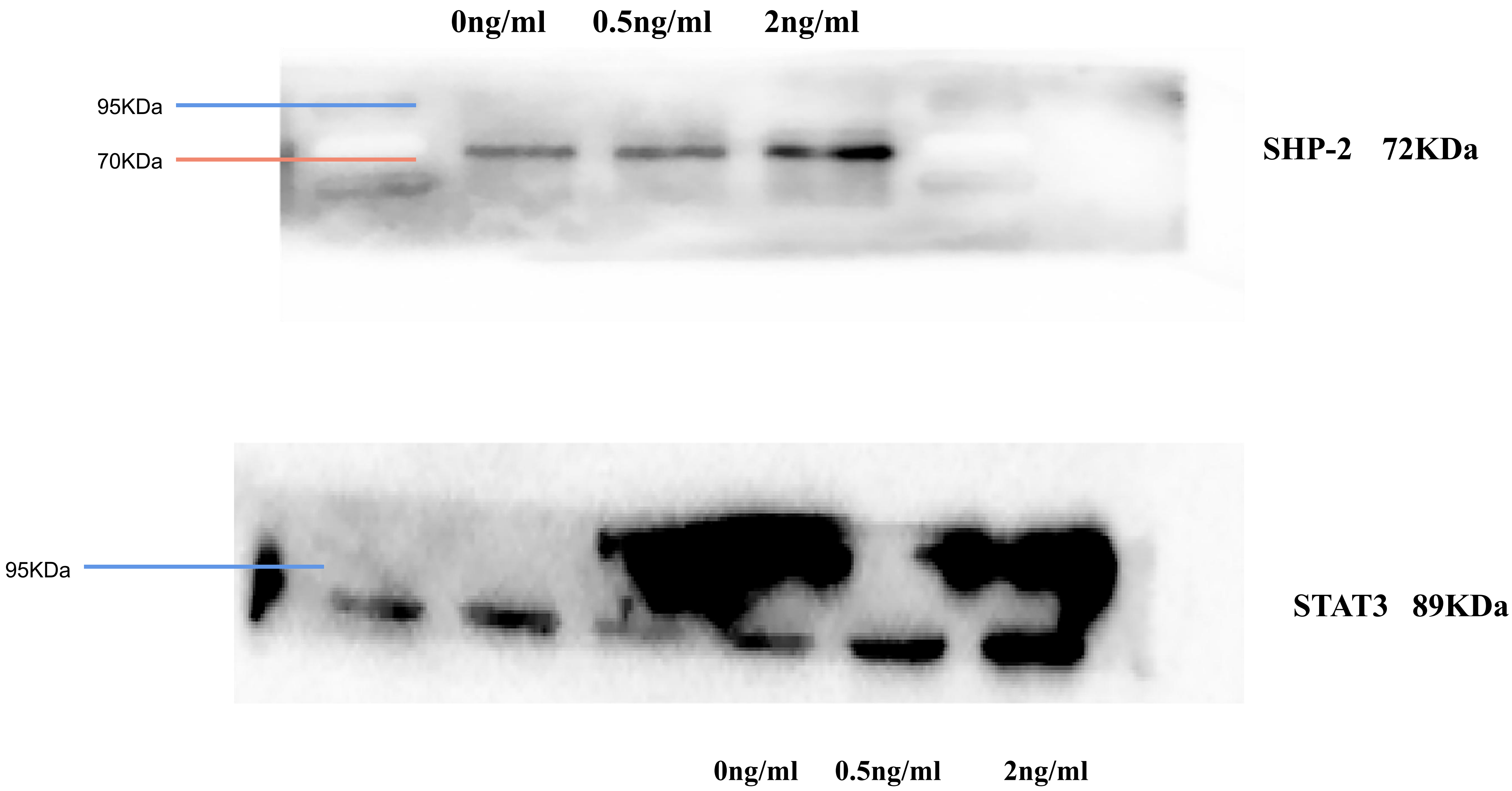

Figure 3A SHP-2 and STAT3 expression in cultured villi added with low concentration of IL-6

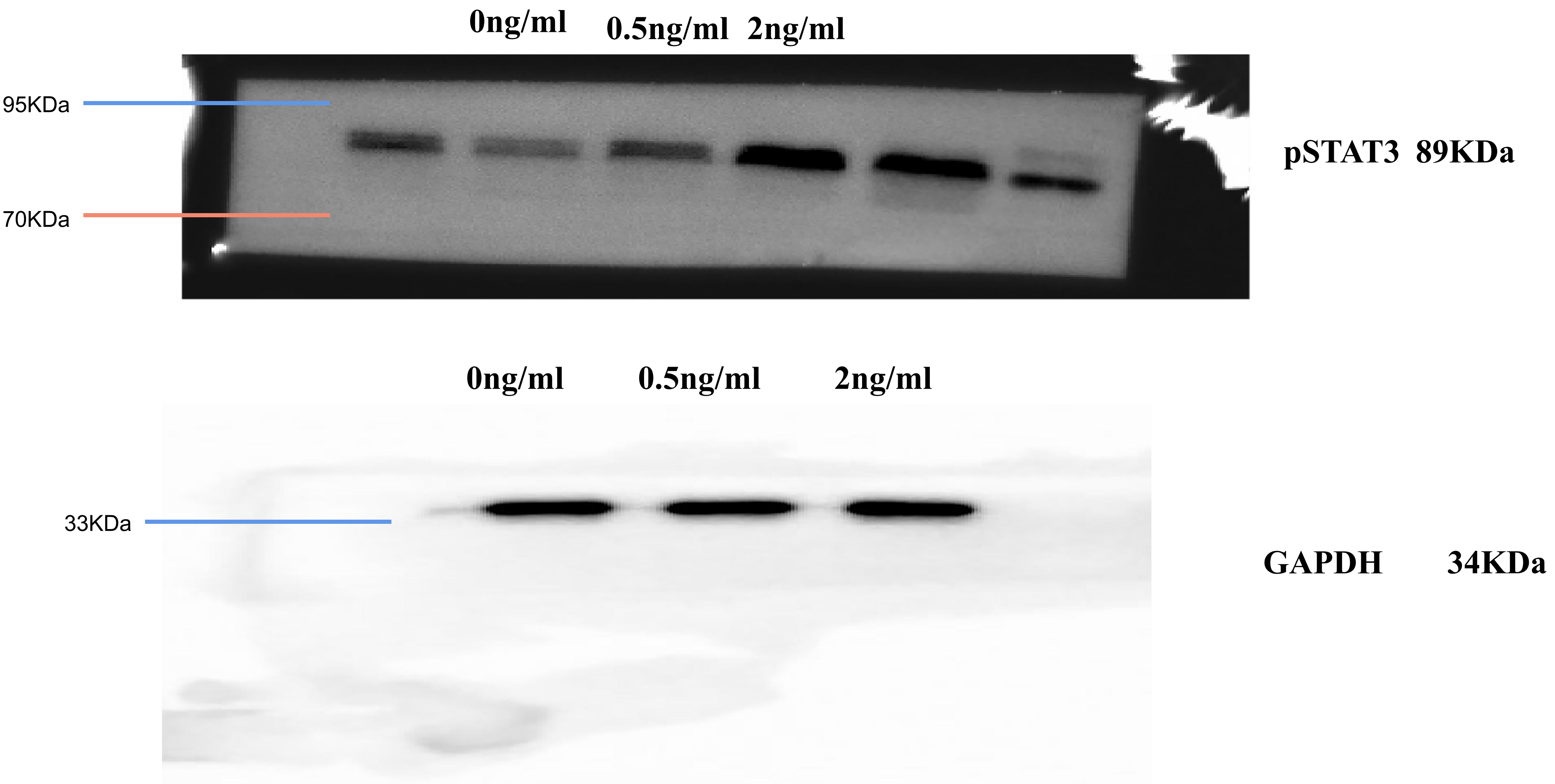

Figure 3A pSTAT3 and GAPDH expression in cultured villi added with low concentration of IL-6

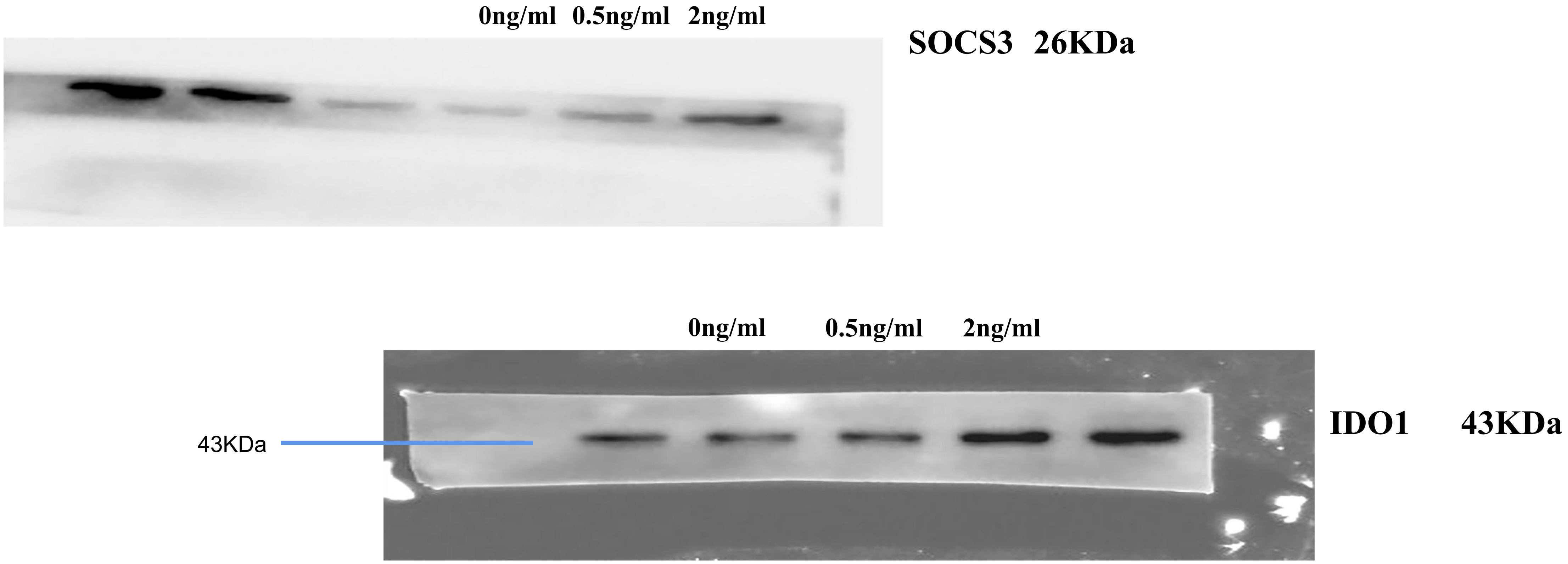

Figure 3E SOCS3 and IDO1 expression in cultured decidua added with low concentration of IL-6

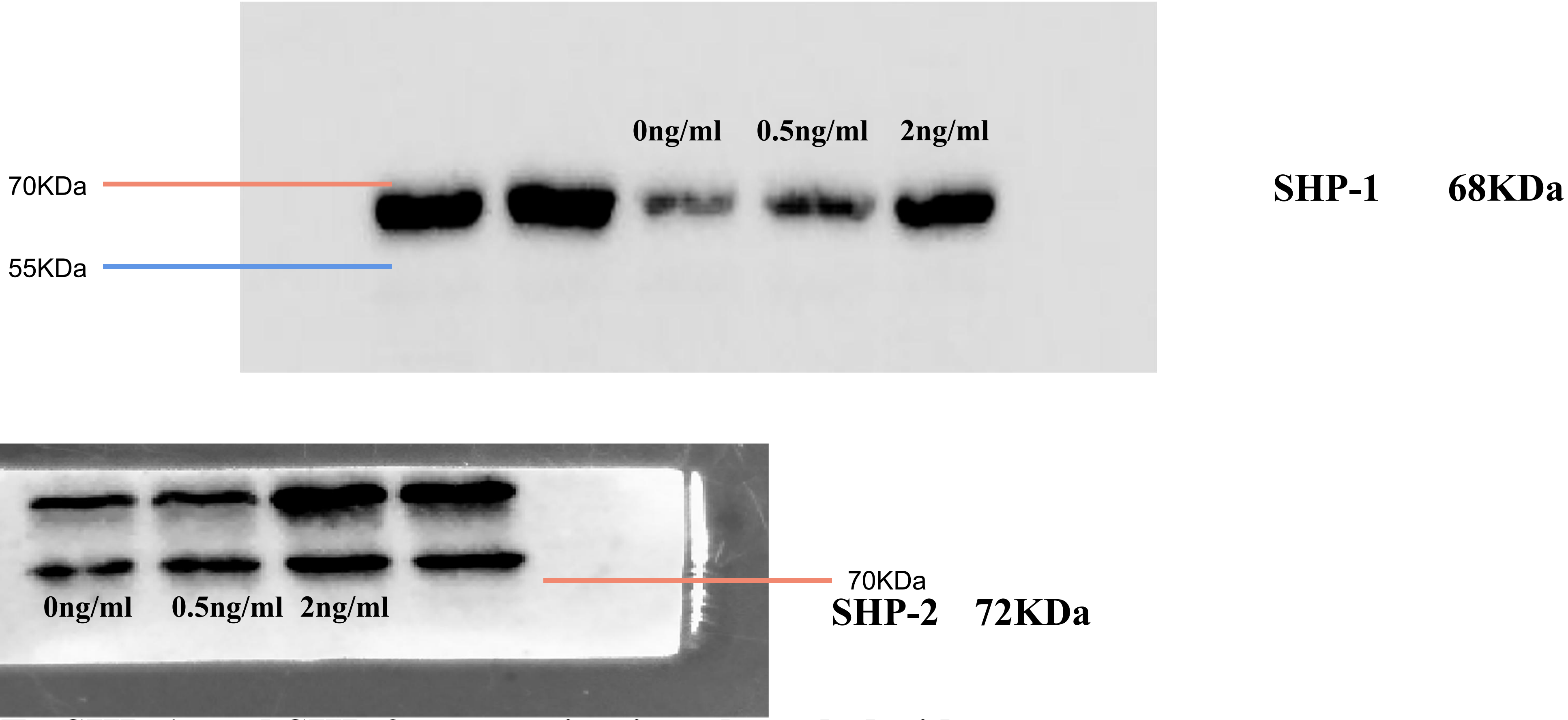

Figure 3E SHP-1 and SHP-2 expression in cultured decidua added with low concentration of IL-6

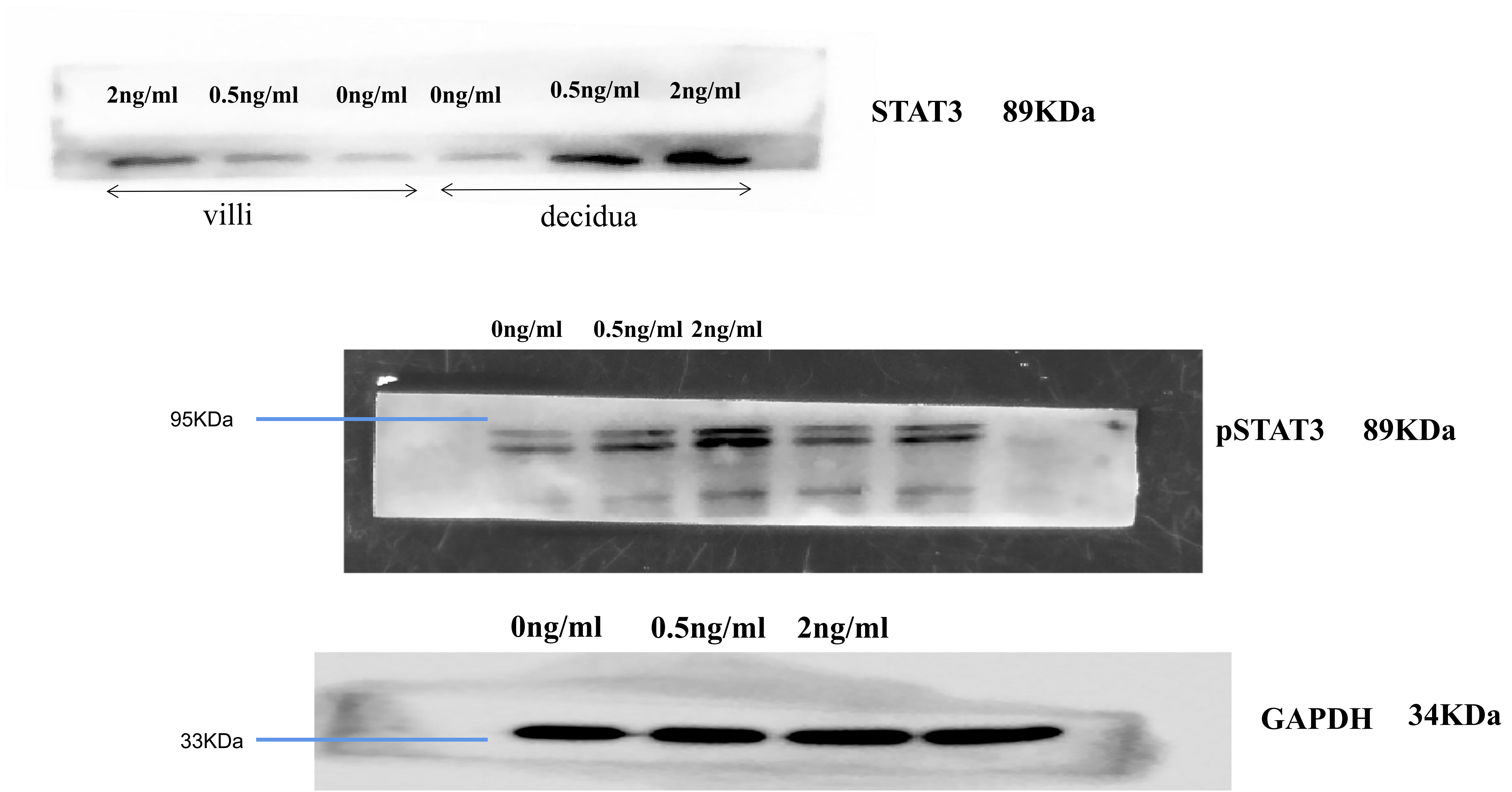

Figure 3E STAT3, pSTAT3 and GAPDH expression in cultured decidua added with low concentration of IL-6

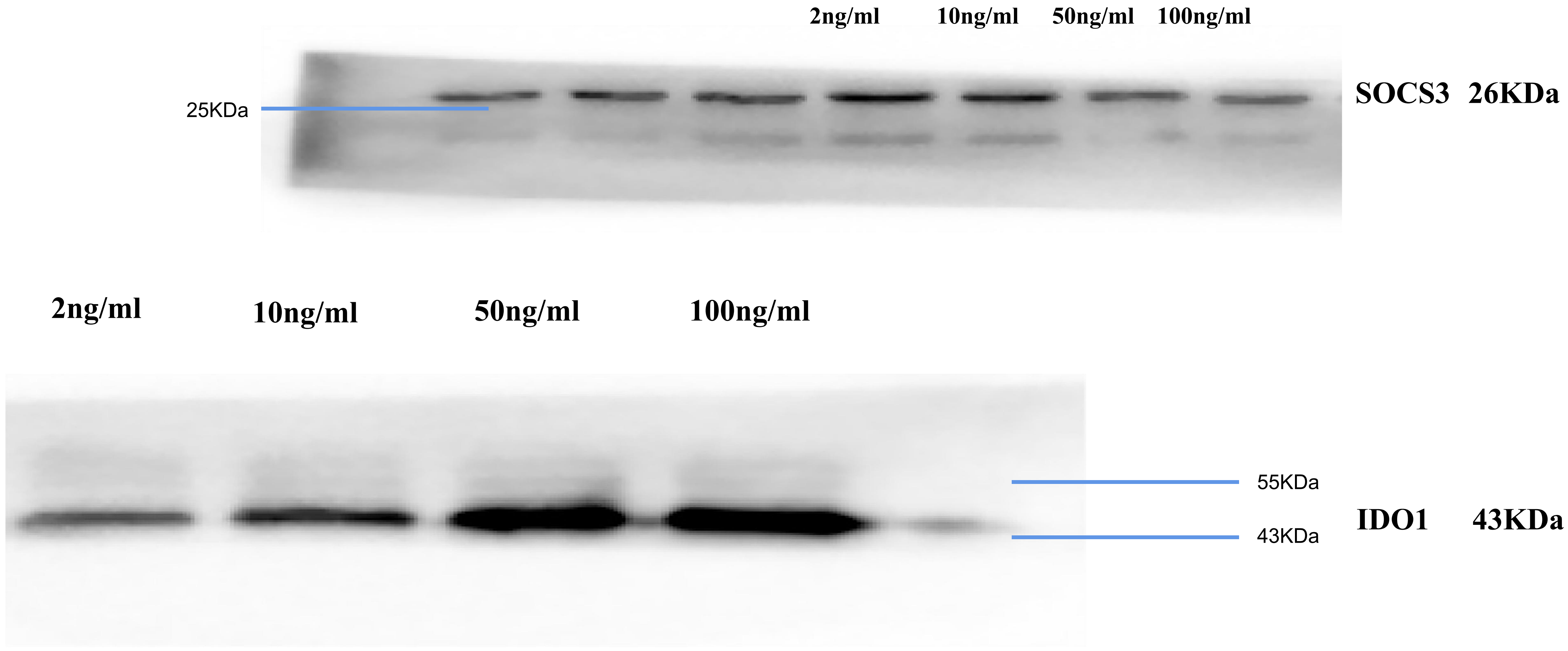

Figure 4A SOCS3 and IDO1 expression in cultured villi added with high concentration of IL-6

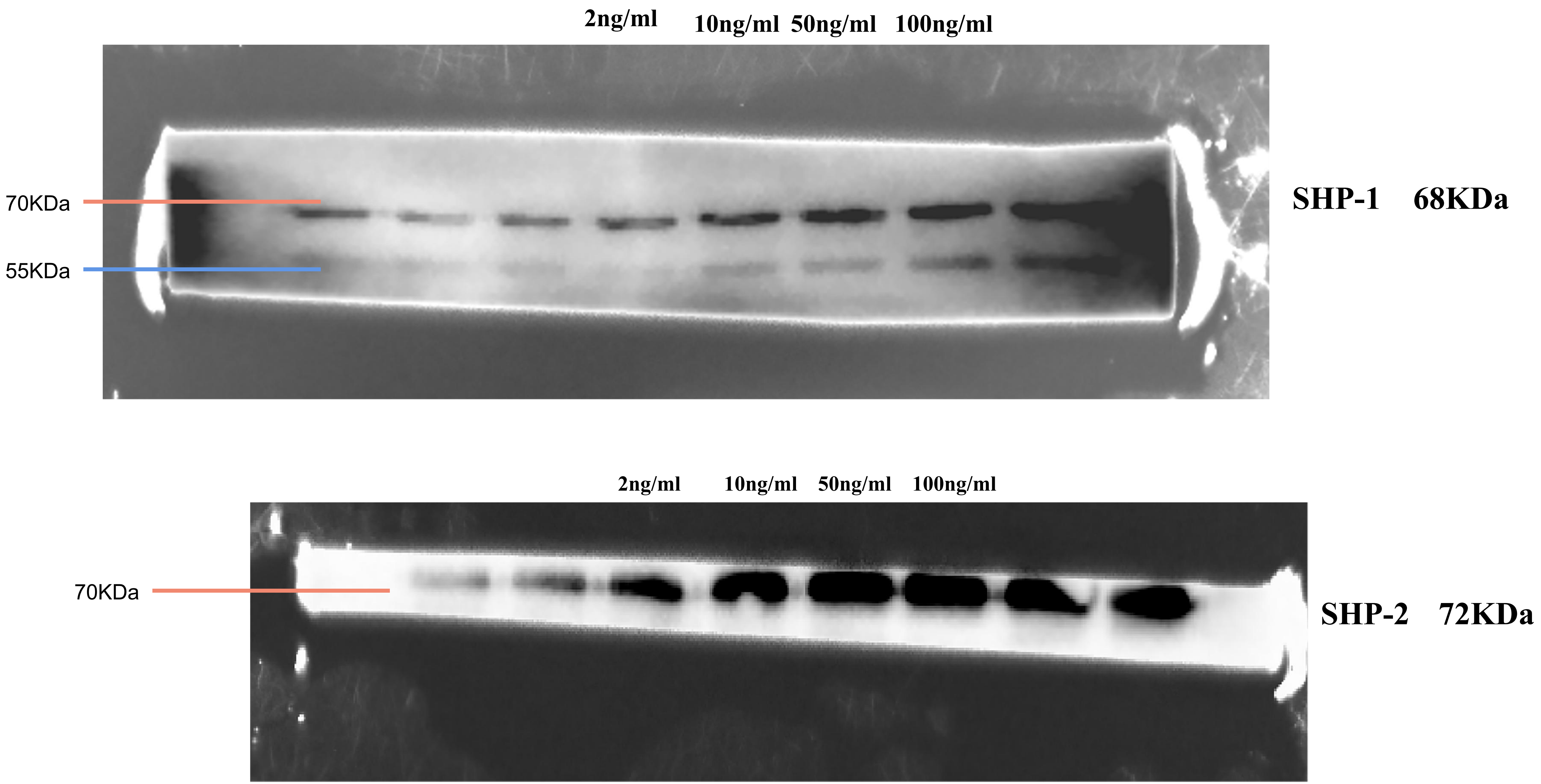

Figure 4A SHP-1 and SHP-2 expression in cultured villi added with high concentration of IL-6

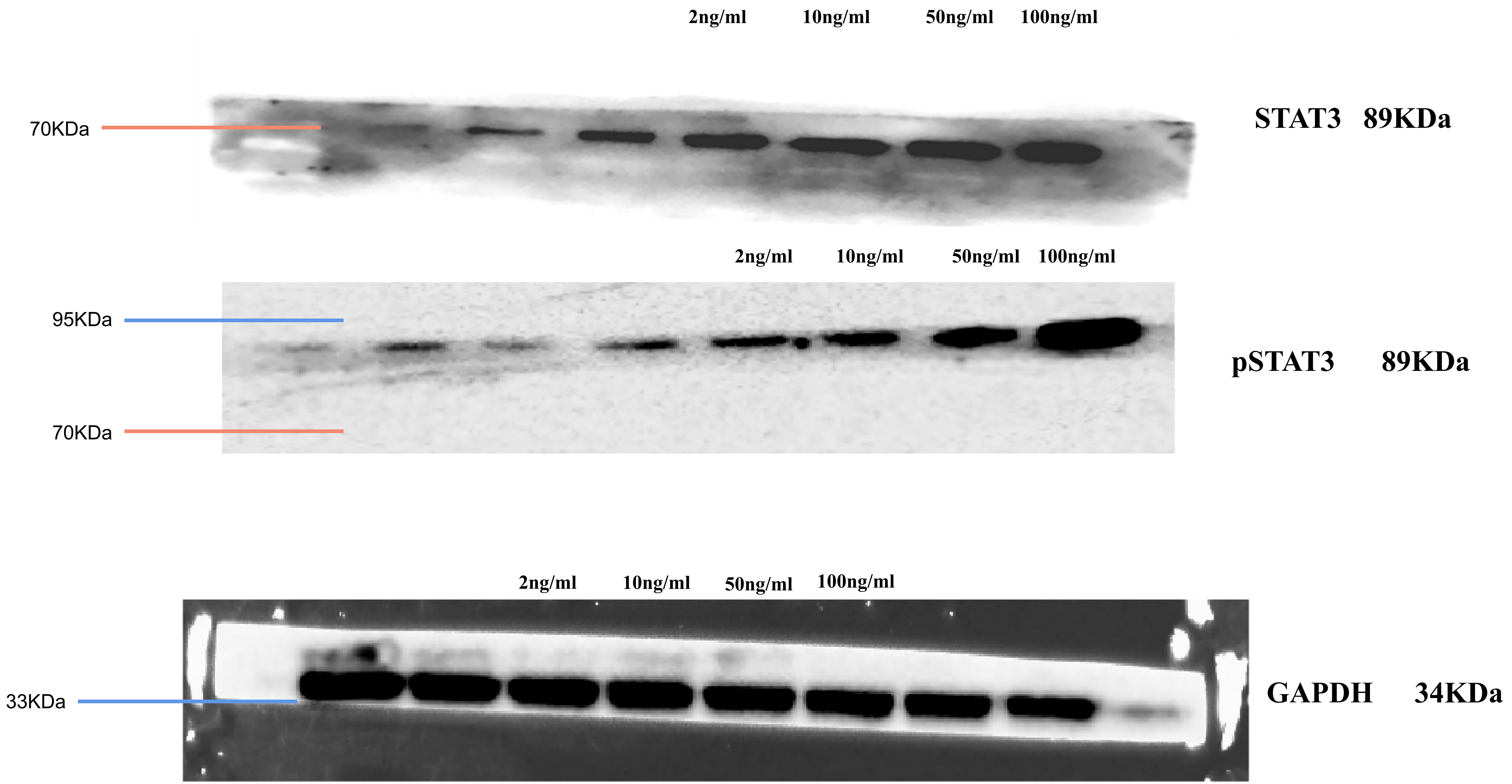

Figure 4A STAT3,pSTAT3 and GAPDH expression in cultured villi added with high concentration of IL-6

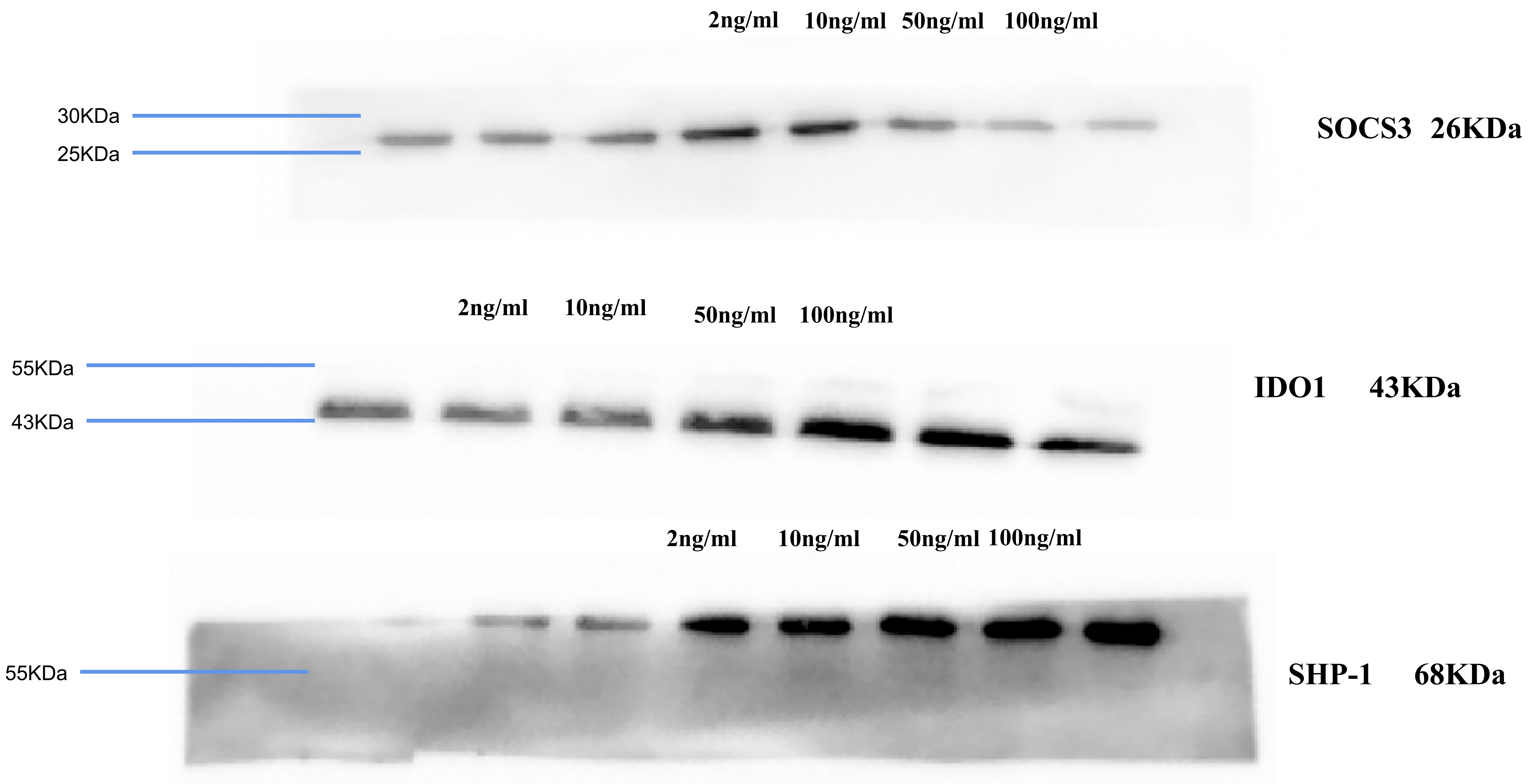

Figure 4E SOCS3, IDO1 and SHP-1 expression in cultured decidua added with high concentration of IL-6

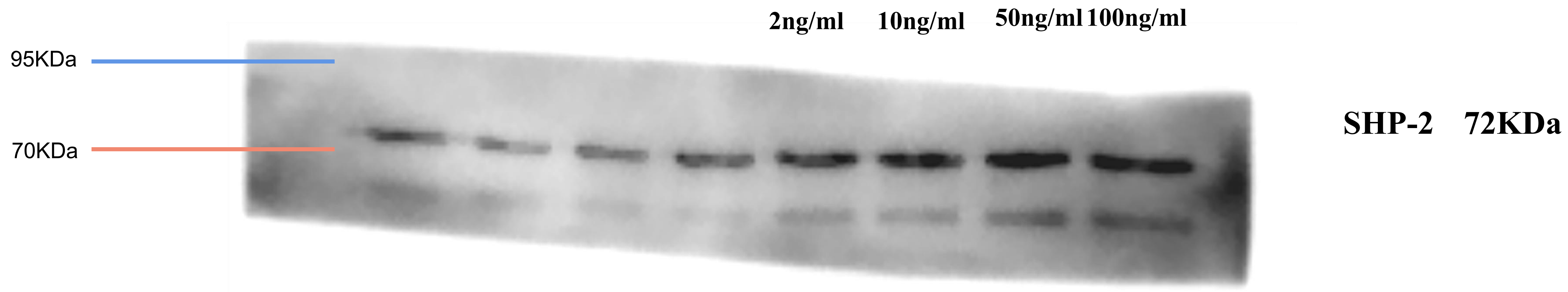

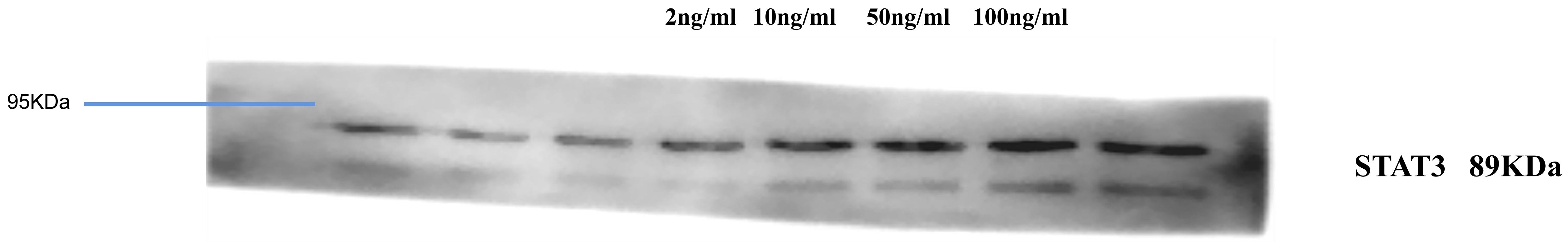

Figure 4E SHP-2 and STAT3 expression in cultured decidua added with high concentration of IL-6

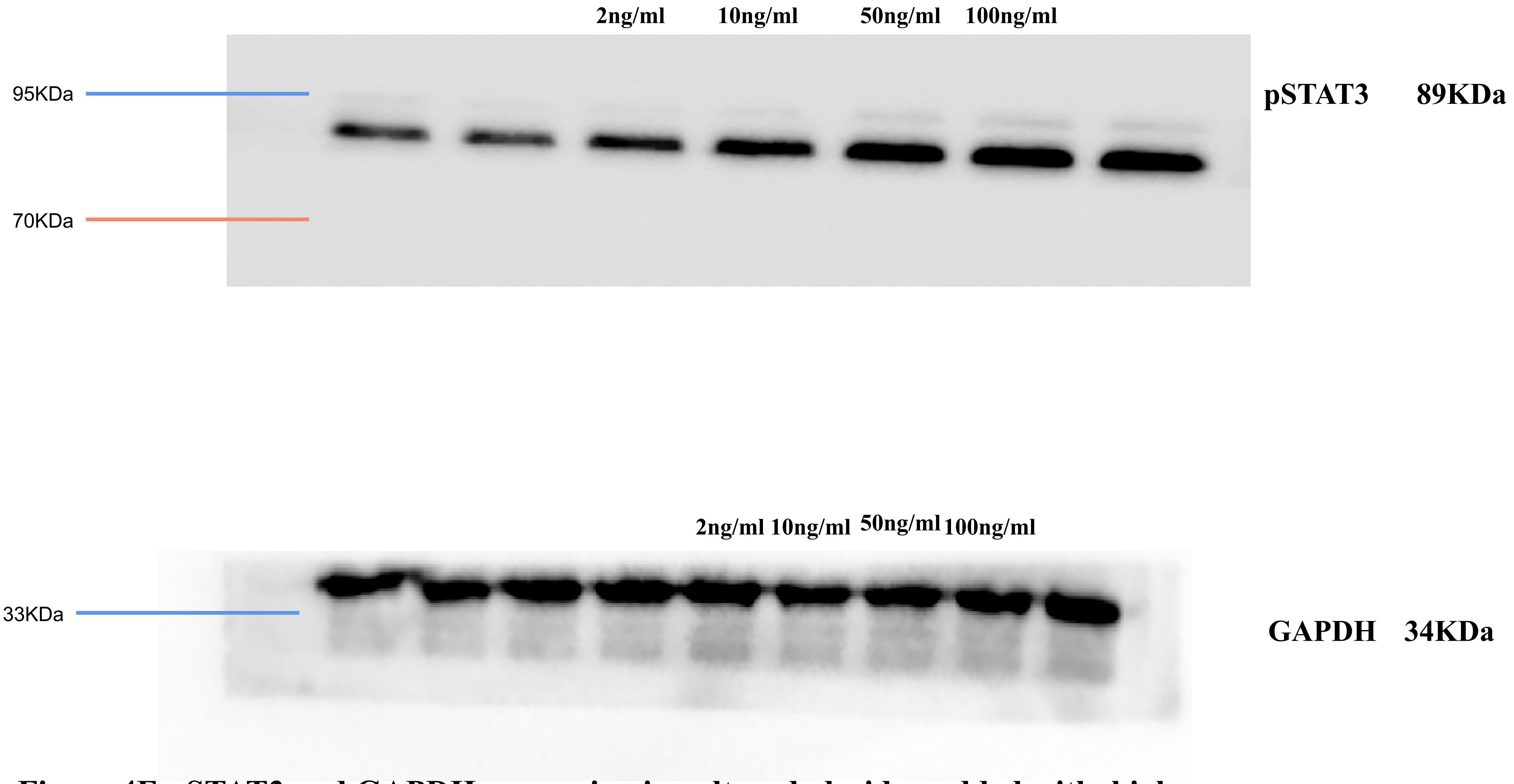

Figure 4E pSTAT3 and GAPDH expression in cultured decidua added with high concentration of IL-6

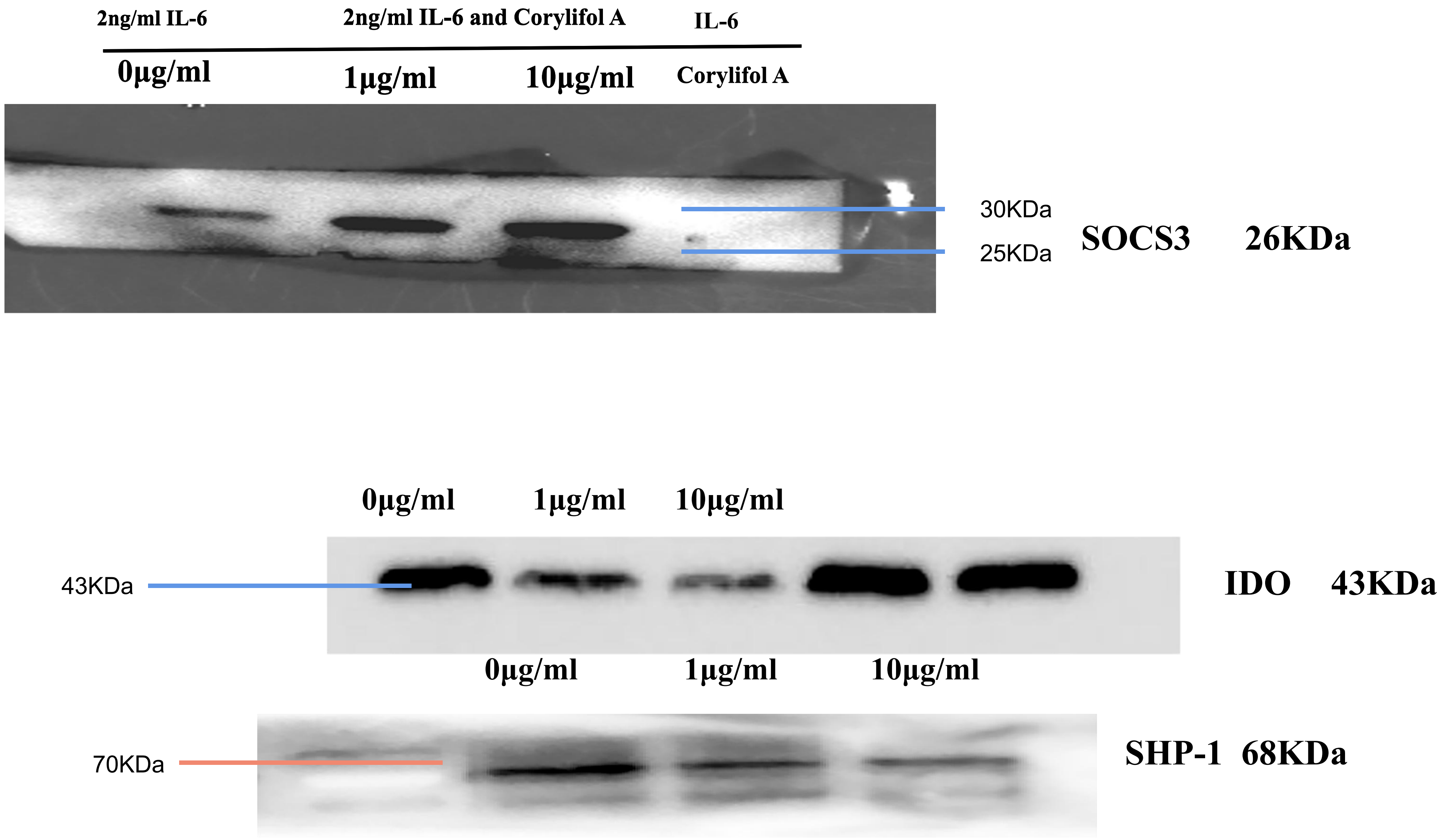

Figure7A SOCS3,IDO, SHP-1,SHP-2 , STAT3 and p-STAT3 expression cultured chorionic villi added with 2ng/ ml IL-6 and Corylifol A

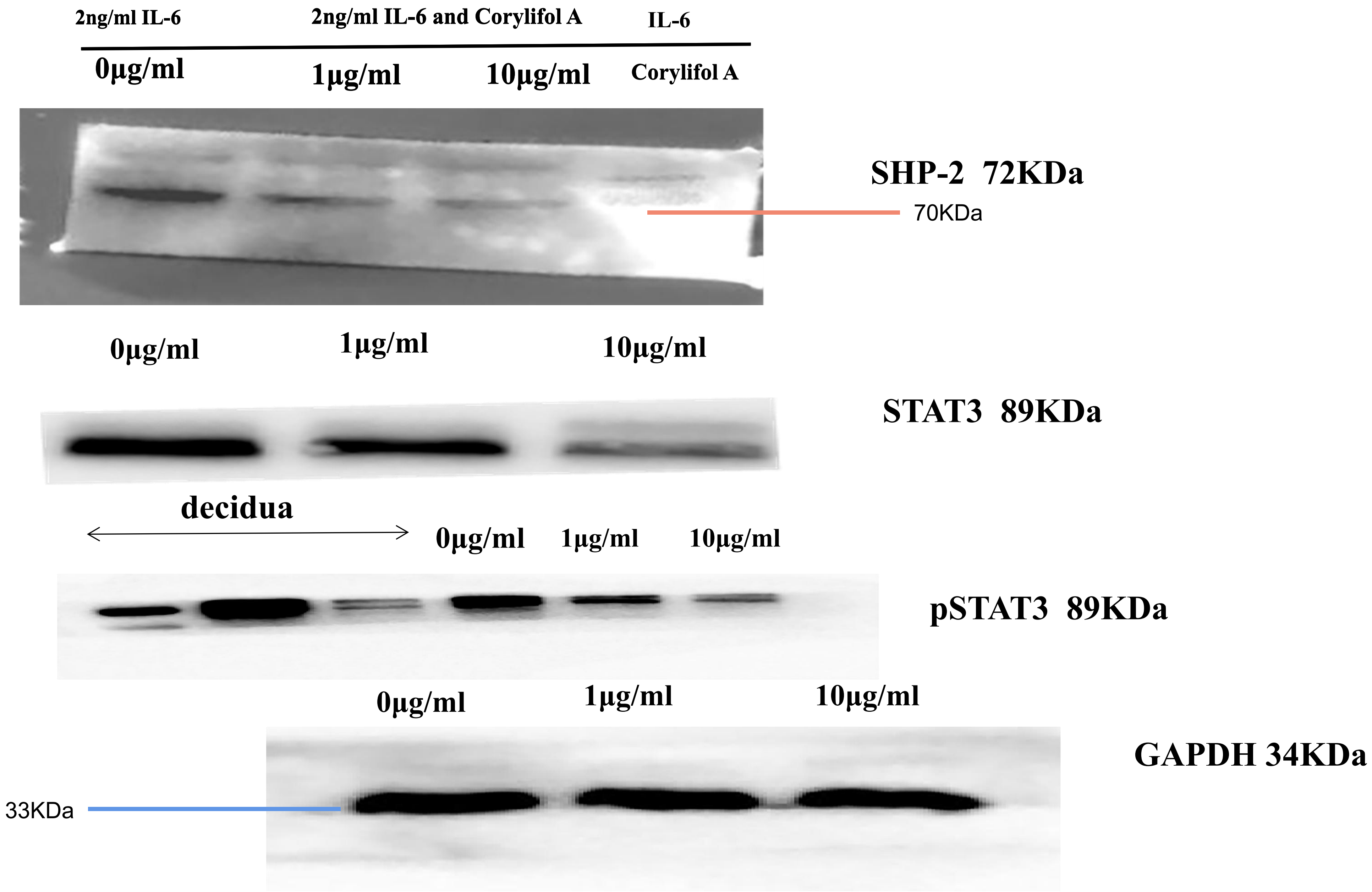

Figure7A SOCS3,IDO, SHP-1,SHP-2 , STAT3 and p-STAT3 expression cultured chorionic villi added with 2ng/ ml IL-6 and Corylifol A

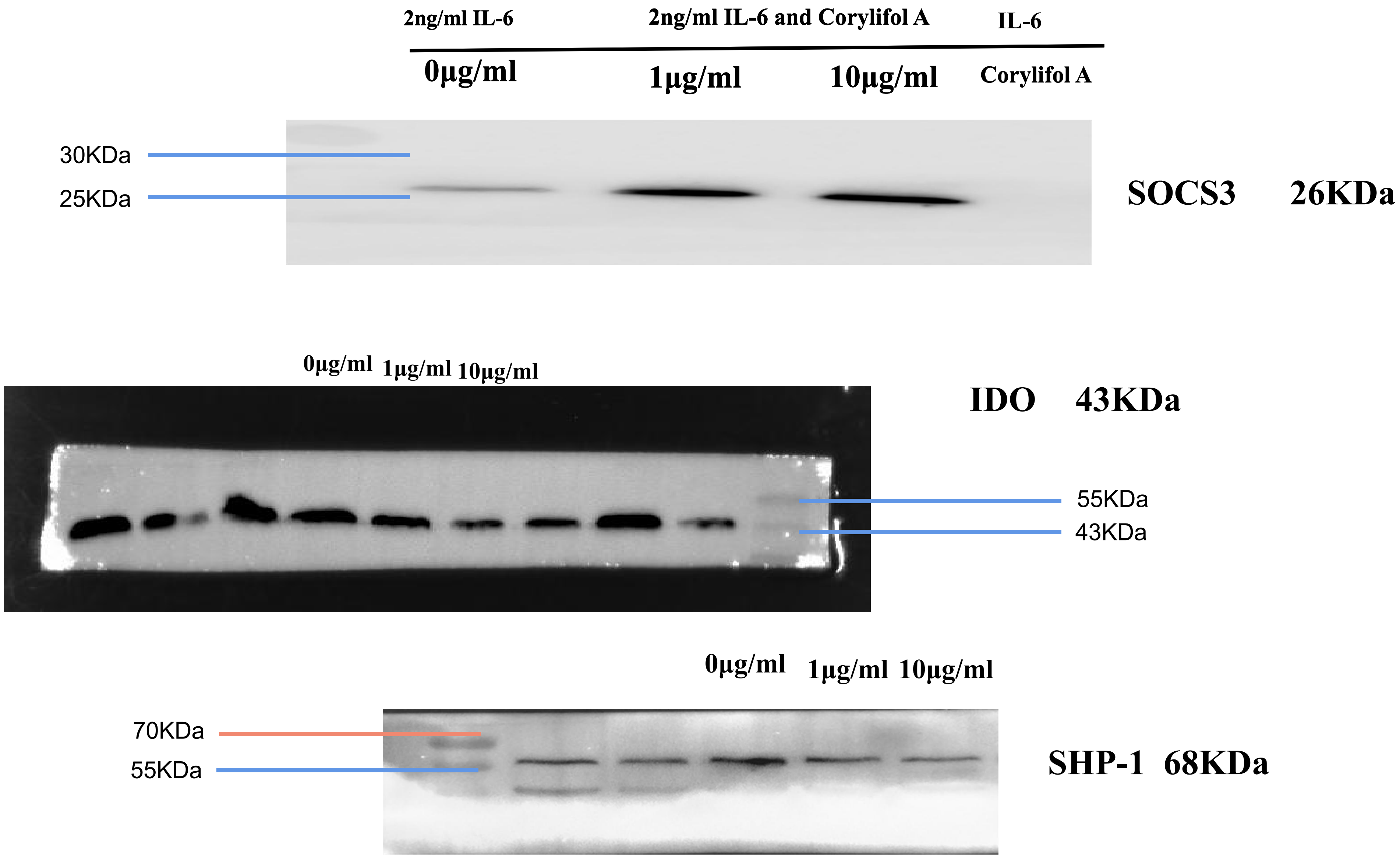

Figure7E SOCS3,IDO, SHP-1,SHP-2 , STAT3 and p-STAT3 expression cultured chorionic villi added with 2ng/ ml IL-6 and Corylifol A

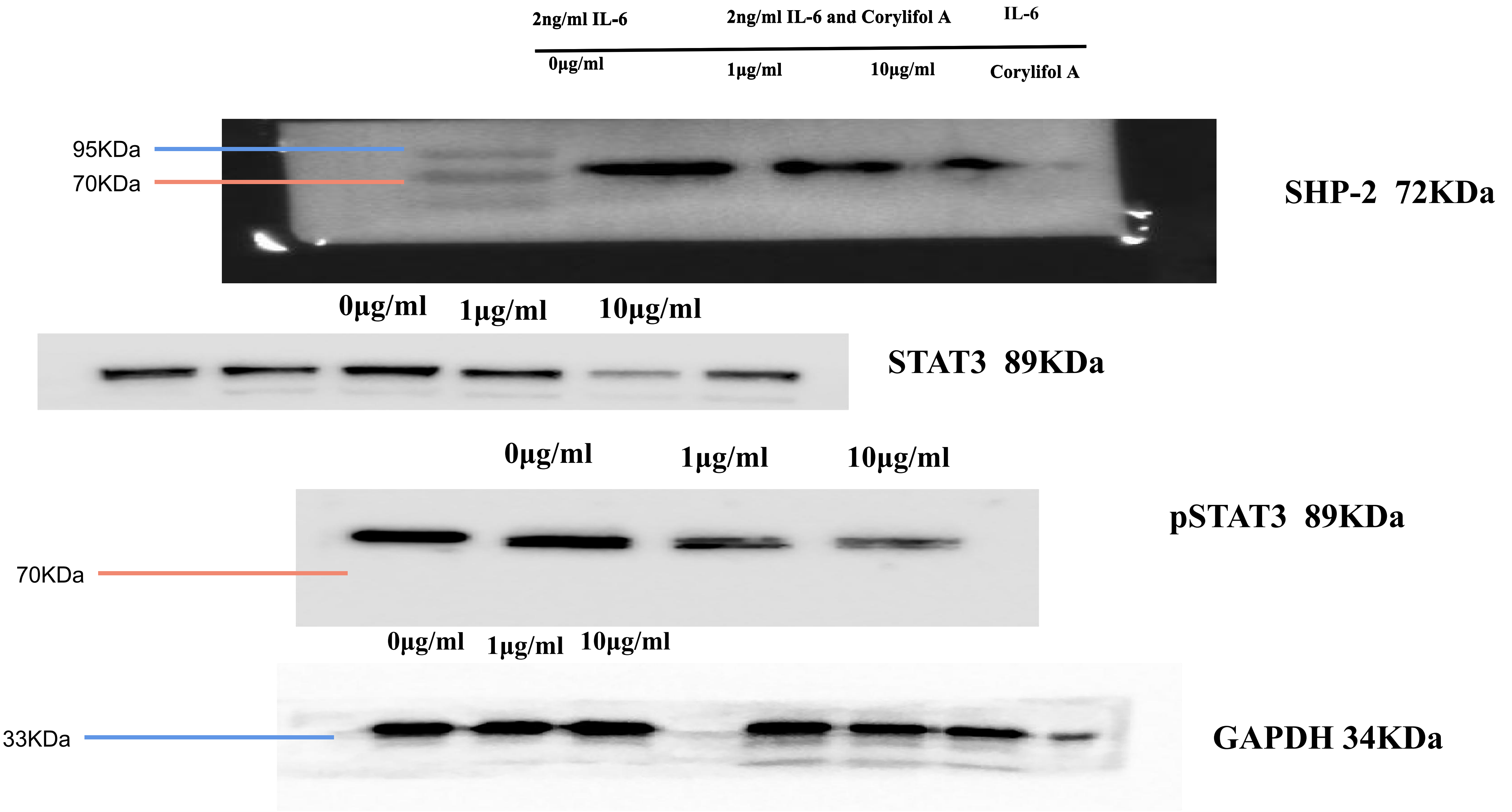

Figure7E SOCS3,IDO, SHP-1,SHP-2 , STAT3 and p-STAT3 expression cultured chorionic villi added with 2ng/ ml IL-6 and Corylifol A
